# Impact of recognition of genetic information related to BMI on changes in physical activity, dietary intake, and blood cholesterol level: a randomized controlled trial

**DOI:** 10.1007/s00394-025-03713-x

**Published:** 2025-05-27

**Authors:** Ga Young Lee, Junghak Lee, Jeong-Han Kim, Kyong-Mee Chung, Sung Nim Han

**Affiliations:** 1 Department of Food and Nutrition, College of Human Ecology, Seoul National University, 1 Gwanak-ro, Gwanak-gu, Seoul, 08826 Republic of Korea; 2 Department of Agricultural Biotechnology and Research Institute of Agriculture and Life Sciences, Seoul National University, Seoul, Republic of Korea; 3 Department of Psychology, Yonsei University, Seoul, Korea; 4 Research Institute of Human Ecology, Seoul National University, Seoul, Republic of Korea

**Keywords:** Genetic information disclosure, BMI, Physical activity, Dietary intake, Cholesterol

## Abstract

**Purpose:**

Genes associated with body mass index (BMI), including *FTO rs9939609,MC4R* rs17782313, and *BDNF* rs6265, may influence BMI and regulate energy metabolism. While previous studies have explored health-related behavior changes, few have investigated both biochemical and behavior changes resulting from perceived genetic risk. This study investigated whether recognizing BMI-related genes affects health-related behaviors and alters blood metabolite levels.

**Methods:**

Normal and overweight adults aged 25–35 years (*n* = 100) were randomly assigned to an intervention group (*n* = 65) informed about BMI-related genetic information (*FTO* rs9939609, *MC4R* rs17782313, *BDNF* rs6265) and an uninformed group (*n* = 35, CON). The intervention group was further divided into Intervention-high risk (IHR, *n* = 36) and intervention-low risk (ILR, *n* = 29) subgroups. Dietary intake and physical activity (PA) were assessed using a 3-day dietary record and the IPAQ-short form. Blood metabolites were analyzed through multivariate analyses to identify significant differences among the groups, with measurements taken at baseline, 3 months, and 6 months.

**Results:**

The IHR group exhibited increased dietary fat and fast foods intake, along with enhanced vigorous and moderate PA. Six metabolites were selected as biomarkers that were distinguishable among groups, and the relative serum cholesterol levels significantly decreased in the IHR group at 3 months.

**Conclusion:**

These results demonstrate that recognizing the BMI-associated genetic risk resulted in a short-term increase in PA but did not improve dietary intake. Increased PA was significantly associated with reduced cholesterol concentration, suggesting the clinical importance of physical activity in the genetically at-risk group.

**Clinical Trial and Study Registration:**

This study was reviewed and approved by the Seoul National University Institutional Review Board (IRB #1901/001–004) and registered on the Clinical Research Information Service (CRIS), KCT0004650 (https://cris.nih.go.kr/cris/search/detailSearch.do /14091, 2020/01/28).

**Supplementary Information:**

The online version contains supplementary material available at 10.1007/s00394-025-03713-x.

## Introduction

Obesity is a multifactorial condition influenced by both genetic and environmental factors [1, 2]. There are various methods to measure body mass, including bioelectrical impedance analysis and dual-energy X-ray absorptiometry, as well as conventional anthropometric measures such as body mass index (BMI) and waist-to-hip ratio [3, 4]. Although BMI does not differentiate between fat and lean mass, it remains a practical tool due to its simplicity and a relatively high correlation with health outcomes, and it is generally used to assess body mass at the population level [5, 6]. Genetic factors also play a significant role in obesity. Single nucleotide polymorphisms such as *FTO* rs9939609, *MC4R* rs17782313, and *BDNF* rs6265, have been consistently associated with elevated body weight and an increased risk of obesity across diverse populations through their roles in appetite regulation, satiety, and energy balance [7–14]. The *FTO* rs9939609 A allele has been linked to increased appetite and a preference for energy-dense foods due to alterations in hypothalamic signaling [15], while the *MC4R* rs17782313 C allele is related to impairment of energy homeostasis by disrupting melanocortin signaling, leading to hyperphagia [16]. Additionally, the *BDNF* rs6265 A allele reduces BDNF expression, negatively affecting satiety and energy balance regulation [17]. Moreover, evidence suggests that combined effects of these variants may synergistically influence obesity susceptibility [18, 19]. Since body weight is modifiable through lifestyle interventions such as improvements of dietary intake and physical activity (PA) [20], providing individuals with genetic information related to increased risk of weight gain may help facilitate in health-related behaviors.

Previous studies have explored the potential for personalized genetic risk information to motivate behavioral changes, such as improvements in diet and PA [21–24]. However, inconsistent results have been reported regarding the impact of genetic information recognition on changes in dietary intake. A systematic literature review of seven studies reported no significant changes in dietary intake following genetic risk perception [21]. Similarly, a review involving 2,322 participants demonstrated that providing personalized nutrition based on genetic information did not improve health-related behaviors [25]. However, a randomized controlled study with 107 healthy individuals showed that those perceiving a low genetic risk for apolipoprotein E had improved dietary fat quality compared to individuals who perceived a high genetic risk or did not perceive any genetic information [23]. Additionally, in a randomized trial involving 103 participants, individuals receiving advice based on genetic risk for *ACE* gene showed a significant reduction in sodium intake compared to those receiving general dietary guidelines without genetic information [24]. Nevertheless, methodological differences in previous studies, such as variations in participant age, health conditions, race, the diversity of genetic information provided, and follow-up duration, resulted in difficulties in obtaining consistent conclusions [26].

Although studies examining changes in PA in response to genetic risk disclosure for obesity have been conducted, existing research has predominantly focused on the *FTO* gene [27, 28]. In one study, awareness of the *FTO* rs9939609 variant was not associated with changes in PA levels [27]. In contrast, another study found that participants who received advice based on their *FTO* gene information exhibited a significant improvement in self-reported PA levels six months after the intervention, compared to those receiving general PA guidelines without genetic information [28]. However, there are still lack of studies that have examined the impact of genetic information related to BMI on changes in PA levels, making it difficult to draw consistent conclusions [27].

Metabolic profiling provides objective and sensitive biomarkers that complement self-reported measures, offering deeper insight into the physiological aspects of lifestyle changes [29]. Among lifestyle factors, PA and dietary intake have been reported as key determinants of metabolic profile alterations as they influence metabolic reaction rates [30, 31]. PA contributes to changes in blood metabolite concentrations by regulating various metabolic pathways, such as lipid and amino acid breakdown [32, 33]. Additionally, daily dietary intake is known to influence blood metabolite levels [31, 34]. Given that genetic risk perception may influence lifestyle behaviors, incorporating metabolomic analysis into such studies can help identify whether and how these behavior changes translate into physiological alternations. However, few studies have utilized omics-based approaches to investigate the physiological effects of genetic information disclosure. This study therefore employed metabolic profiling to assess both self-reported behavioral changes and underlying biological responses, providing a more comprehensive evaluation of the impact of genetic information disclosure.

In this study, we hypothesized that the perception of genetic risk related to BMI would lead to improvements in health-related behaviors, such as PA and dietary intake. To test this hypothesis, we aimed to examine the impact of the recognition of genetic risk for *FTO* rs9939609, *MC4R* rs17782313, and *BDNF* rs6265 on changes in PA and dietary intake, as well as the resulting changes in blood metabolite concentrations.

## Methods

### Study design and participants

This study was a parallel-group randomized controlled trial with a 2:1 ratio of participants in the intervention to the control groups. Adults aged 25‒35 years with a body mass index (BMI) of 18.5–25 kg/m^2^ (ranging from normal weight to individuals at risk of overweight, according to the WHO Asia-Pacific classification of BMI) were included in this study. Individuals with diseases such as cancer, diabetes, kidney disease, cardiovascular or pulmonary conditions, as well as those taking medications for mental health disorders, were excluded due to their likelihood of having made lifestyle modifications. Recruitment was carried out from December 2018 to January 2019 at Seoul National University, and the study was conducted from February 2019 to September 2019. Of 111 screened candidates, 100 eligible participants provided informed consent. This study was approved by the Seoul National University Institutional Review Board (IRB #1901/001–004) and registered with the Clinical Research Information Service (CRIS, KCT0004650).

Participants were randomly assigned to either the intervention (*n* = 65) or control group (CON, *n* = 35), based on a previous study that demonstrated varying responses when individuals became aware of their genetic test results compared to those in the control group [35]. Randomization was conducted using a random list generated by the PROC survey-select in SAS version 9.4 (SAS Institute, Cary, NC, USA), with concealment from participants until the baseline survey completion. All participants were informed of their group assignments at the beginning of the intervention, while the outcome assessor was blinded to group assignments using randomly assigned IDs and questionnaires.

### Intervention

The intervention group (*n* = 65) was divided into subgroups based on genetic risk score (GS) linked to BMI-related genetic variants, including *FTO* rs9939609, *MC4R* rs17782313, and *BDNF* rs6265, as reported in the test results. The GS was calculated by combining risk alleles and effect size from previous Genome-Wide Association Studies (GWAS) (*FTO* gene: 1.27 [36], *MC4R* gene: 1.24 [12], *BDNF* gene: 1.16 [37]. Participants were categorized into three groups including CON group (control, unaware group, *n* = 35), ILR group (informed-low risk, GS = 0-2.3 points, *n* = 29), and IHR group (informed-high risk, GS = 2.4–6.1 points, *n* = 36). Furthermore, the control group was subdivided into CLR group (control-low risk, GS = 0-2.3 points, *n* = 18) and CHR group (control-high risk, GS = 2.4–4.9 points, *n* = 17) based on the level of risk alleles. Participants in the IHR and ILR groups received individual reports of their genetic test results at the start of the intervention. These reports included SNP results (good/borderline risk/caution) along with brief explanations of the implications of carrying risk alleles. In contrast, participants in the CON group were provided with their genetic test results after the study was completed.

### Genotyping

Genotyping was conducted by the TheragenEtex Bio Institute (TheragenEtex Inc., Suwon, Republic of Korea) according to the Affymetrix Axiom Custom Assay Plate Protocol using a Theragen Precision Medicine Research Array chip. The genetic test results for genes related to BMI are present in Supplementary Table 1.

### Study outcomes

The primary outcome of the study was the changes in health-related behaviors, including dietary intake assessed through a 3-day dietary record and PA evaluated using the IPAQ-short form between baseline to each follow-up assessment. The secondary outcome was the association between the health-related behaviors, including dietary intake and PA, and the blood metabolite levels. 

### Anthropometric assessment

Anthropometric measurements were conducted at the baseline and the 3-, and 6-month follow-ups. Body weight (kg) and height (cm) were assessed using a digital stadiometer (BSM 330, Biospace, Korea). Body fat mass (kg) and skeletal muscle mass (kg) were measured using a bioelectrical impedance analyzer (Inbody 720, Biospace, Seoul, Korea).

### Assessment of dietary intake

The intake of energy and nutrients was measured at the baseline and the 3-, and 6-month follow-ups using a 3-day dietary record, including two days on a weekday and one day on a weekend. Nutrient analysis was conducted using the Computer Aided Nutritional Analysis Program (CAN-Pro) 5.0 (Web ver. The Korean Nutrition Society, Korea). The food consumed by the participants was classified into seven food groups, including fat and oil group and beverage and fast food group, in addition to the five standard food groups classified based on the 2020 Dietary Reference Intakes for Koreans [38]. Furthermore, they were subdivided into 24 food groups based on the similarity of nutrients; eight grain categories, six protein categories, three vegetable categories, two fruit categories, one dairy category, one fat category, two added sugar categories, and one fast food category.

### PA assessment using International Physical Activity Questionnaire-Short form and wearable devices

The participants completed self-reported surveys at the baseline and the 3-, and 6-month follow-ups through the online survey (http://www.surveymonkey.com) based on the International Physical Activity Questionnaire-short form (IPAQ-SF) (Supplementary Table 2). Participants were asked to record the frequency, duration, and intensity of their activities. Data from the IPAQ-SF were converted to continuous variables according to the IPAQ-SF analysis guideline, defined as Metabolic Equivalent of Task (MET) value-hr per week. Each MET score was calculated by multiplying MET values (8.0 for Vigorous activity, 4.0 for Moderate activity, 3.3 for Walking activity) with the duration of PA (hr/week). Additionally, sedentary time was measured using the Fitbit Charge 3 (Fitbit Inc., San Francisco, CA, USA). Participants wore the device for the 10 days before each visit, with PA levels assessed using the sum of values over 7 consecutive days (hr/week), excluding days with device recognition errors.

### Measurement of metabolites by GC-MS/MS

Peripheral blood collected at the Seoul National University Health Care Center was immediately centrifuged at 2,000 rpm for 20 min to obtain serum samples, and kept at -80 °C until analysis. Metabolites were analyzed using GC-MS/MS (Shimadzu GCMS-TQ8040) with multiple reaction monitoring ions using 50 µL of serum at the Pesticide Chemistry and Toxicology laboratory of the Department of Agricultural Biotechnology of Seoul National University. The conditions for the analysis of serum metabolites by using GC-MS/MS was described in a previous study [39]. A total of 304 metabolites were measured, including monosaccharides, amino acids, and fatty acids. Out of these, 93 metabolites combining the forms of isomers or various derivatives were statistically analyzed; 20 amino acids, 28 carbohydrates, 1 ester, 1 indole, 2 amides, 1 amine, 1 alcohol, 8 fatty acids, 1 glyceride, 2 steroids, 5 nucleotides, 3 phenols, 1 tocopherol, 19 organic acids. The peak area of the metabolites was compared with 2-isopropylmatic acid as an internal standard to calculate the relative area under the curve for each metabolite.

## Statistical analysis

The sample size was estimated from a similar study [23], calculating 55 per group based on a 1.9-point difference in dietary fat quality, standard deviation of 3.6, and 80% power. Considering follow-up losses, 65 participants were recruited. The last observation carried forward was used for participants who did not complete the intervention period. Differences in changes in the anthropometric data, PA, nutrient intake, and metabolites among the groups were analyzed using One-way ANOVA tests for normally distributed data and Kruskal-Wallis tests for skewed data, with Bonferroni-correction multiple comparison test for post-hoc analysis. Changes to the follow-ups from the baseline were tested using paired *t-*tests for normally distributed data and Wilcoxon signed-rank tests for skewed data. A Spearman correlation was used to determine the association between parameters. Linear regression was used to examine the association between PA and dietary intake and metabolites concentrations, adjusting for age (years), sex, BMI (kg/m^2^), and nutrient intake (g/d). Repeated measure ANCOVA was used to determine significant changes in the serum cysteine levels over time among groups, controlling for confounders, such as age, sex, BMI, protein intake, and TPA. Analyses were performed using SPSS Statistics version 26 (IBM SPSS Statistics, Chicago, IL, USA) with significance set at *P* < 0.05 in two-sided tests.

Multivariate analysis of metabolites was conducted using SIMCA- P + software (version 17.0, Umetrics, Umea, Sweden). To confirm the separation of metabolites among the groups, a principal component analysis (PCA) and partial least squares-discriminant analysis (PLS-DA) were performed, calculating R2X, R2Y, and Q2 parameters. Orthogonal partial least squares-discriminant analysis (OPLS-DA) was conducted to further distinguish the differences of the metabolites within each group, with models validated by 1000 permutation tests. The S-Plot and VIP (variable importance in the projection) were generated in OPLS-DA to select significant metabolites, using cutoff value of │P│ ≥ 0.05 and │P(corr)│≥ 0.5 for S-plot, and VIP scores > 1 with standard errors < 1 [40]. To differentiate metabolites contributing to group differences, Shared and unique structures plot (SUS-Plot) was conducted.

## Results

### Participant characteristics

**Fig. 1:**
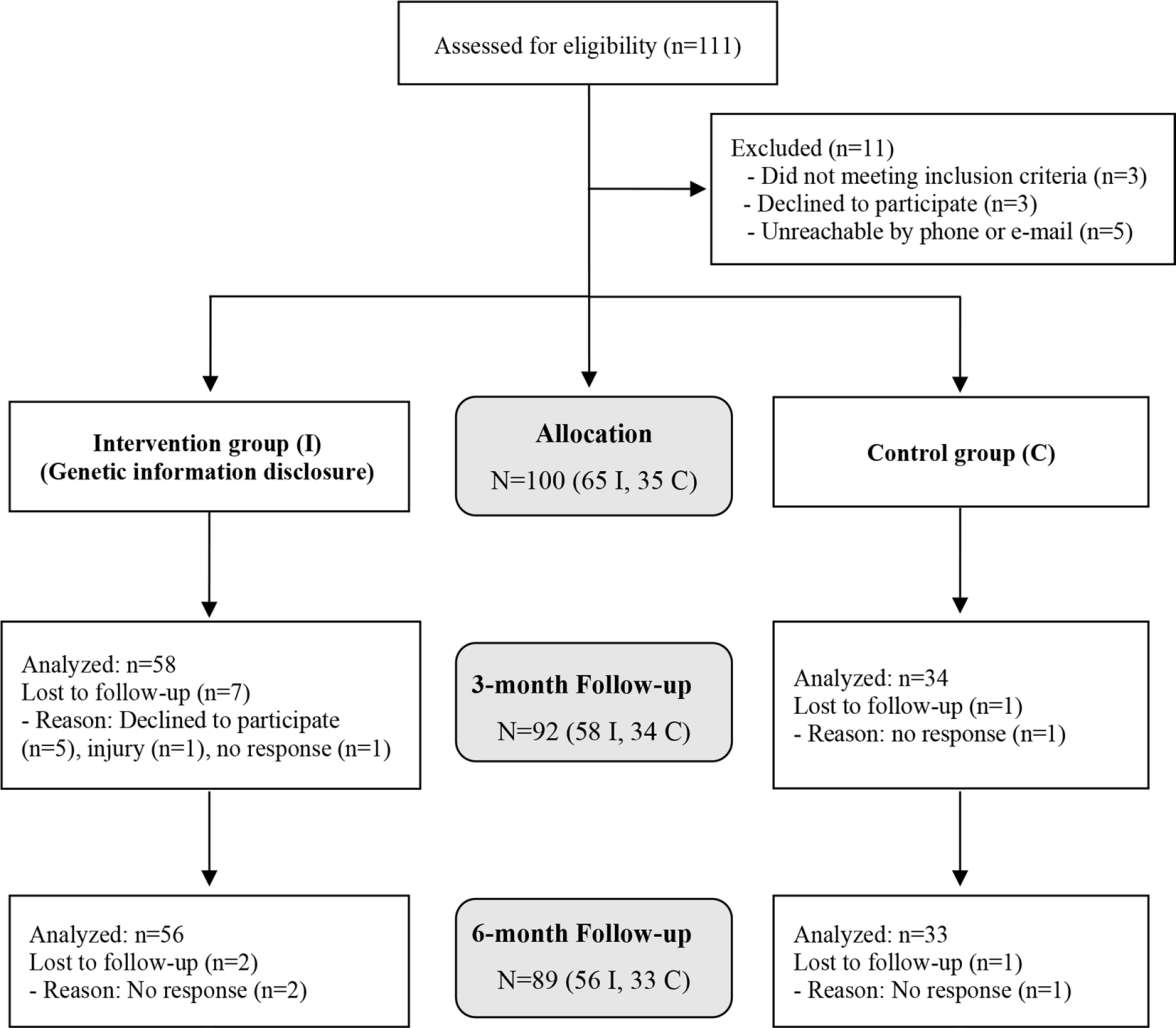
Consolidated standards of reporting trials (CONSORT) flow of the study

Of the 100 participants, 56 Intervention group and 33 CON group completed the trial with an overall retention rate of 89% (Fig. 1). The baseline characteristics of the participants are presented in Table 1. The average age of the participants was 28.1 years, and the mean body mass index (BMI) was 22.3 kg/m^2^. Age and BMI were significantly higher in the IHR group compared to the ILR group, while body fat mass was larger in the CHR group compared to the ILR group. No significant differences were observed among the groups in terms of sex, skeletal muscle mass, total PA levels, and the intake of energy and macronutrients.

### Comparison based on genetic risk levels

Total PA (MET-hr/week), energy intake (kcal/d), and the intakes of carbohydrate, protein, and fat (% energy/d) showed no significant differences between the individuals with high genetic risk and those with low genetic risk based on the genetic scores of the *FTO*,* MC4R*, and *BDNF* genes. However, individuals at high genetic risk had significantly higher age and BMI than those at low genetic risk (Supplementary Table 3).

### Changes in anthropometric measurements

There were no significant differences in changes in anthropometric measurements among the CON, ILR, and IHR groups. In male participants, body fat mass (kg) decreased at 3 months in both the ILR and IHR groups, and only in the IHR group at 6 months. In females, body fat mass decreased in the CON and ILR groups at both 3 months and 6 months, while it decreased only at 3 months in the IHR group (Supplementary Table 4).

### Changes in food and nutrient intake

Total fat intake relative to total energy intake (% energy/d) at 3 months tended to be higher in the IHR group compared to the CON group (*p* = 0.059). A significant difference in change in fat intake (% energy/d) was observed between the IHR group and the CON group, with an increase in the IHR group and a decrease in the CON group (Independent t-test, *p* = 0.031). The changes in saturated fatty acids (g), monounsaturated fatty acids (g), and polyunsaturated fatty acids (g) were not significantly different among the groups at both the 3 months and 6 months. Additionally, the change in the consumption of sugar-sweetened beverages (SSBs, g/d) was significantly higher in the IHR group at both 3 and 6 months compared to the CON group. SSB intake significantly decreased from baseline at both 3 and 6 months in the CON group, whereas an increase was observed in the IHR group at 6 months (Table 2). When comparing the intake of micronutrients, vitamin D intake (µg/d) significantly decreased in the IHR group at 3 months compared to baseline, while there were no significant changes in the CON and ILR groups. Additionally, vitamin C intake (mg/d) also significantly decreased in the IHR group at both 3- and 6 months, with no significant differences observed in the CON and the ILR groups. On the other hand, the intake of dietary fiber (mg/d) significantly decreased in the IHR group at the 6 months, with no significant differences observed in the CON and ILR groups. (Supplementary Table 5).

When classified into four groups based on genetic risk, no significant differences were found among the groups in the changes in total fat intake relative to total energy intake (% energy/d) or in fast food consumption (g/d). However, the change in SSB consumption tended to be higher in the IHR group compared to the CHR and CLR groups (*p* = 0.064), and at 6 months, this change was significantly larger in the IHR group compared to the CLR and CHR groups (Supplementary Fig. 1).

### Changes in PA

**Fig. 2:**
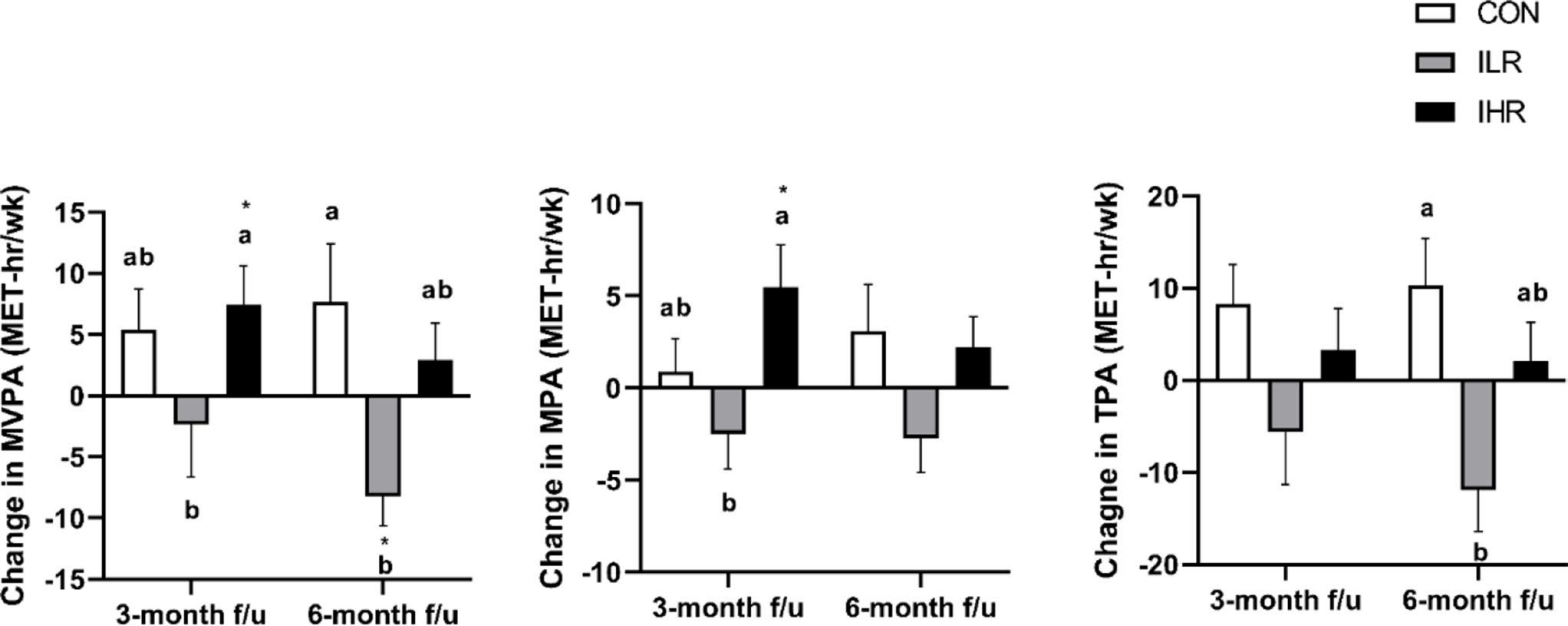
Comparison of the physical activity measurements among the CON, ILR, and IHR groups ^1, 2, 3. 1^ Change = measurements at follow-up time-point– measurements at baseline. ^2^ P value was calculated using one-way ANOVA and Kruskal-Wallis tests followed by Bonferroni-correction for multiple comparisons to determine the differences in PA changes among the CON, ILR, and IHR groups. ^3^ The asterisk indicates significant differences (*P* < 0.05) in the physical activity measurements from the baseline to the follow-up time point.

The levels of moderate to vigorous PA (MVPA) and moderate PA (MPA) (MET-hr/week) were significantly increased in the IHR group at 3 months compared to baseline, whereas no significant changes were observed in the CON and the ILR group. The magnitude of change in both MVPA and MPA was significantly larger in the IHR group compared to the ILR group. At 6 months, the magnitude of change in MVPA and TPA was significantly greater in the CON group than in the ILR group (Fig. 2).

### Metabolomic distinction using multivariate analyses

The PLS-DA analysis of metabolite profiles at 3 months post-intervention showed no distinct separation of metabolites among the groups (Supplementary Fig. 2). PCA was performed to determine the distribution of metabolites within each group, and the metabolites were classified into two clusters per group (Supplementary Fig. 3). To identify specific metabolites contributing to the separation within each group, an OPLS-DA model was developed based on the PCA score plot, and its reliability confirmed through 1000-permutation cross-validation (Supplementary Fig. 4). Significant metabolites were selected using S-plot and VIP analyses, applying criteria of │P│≥0.05, │P (corr)│≥0.5, VIP scores > 1, and standard errors < 1 (Supplementary Fig. 5). Despite identifying several candidate metabolites for each group, the ANOVA analysis during cross-validation showed no significant differences in relative metabolite concentrations among the groups.

**Fig. 3:**
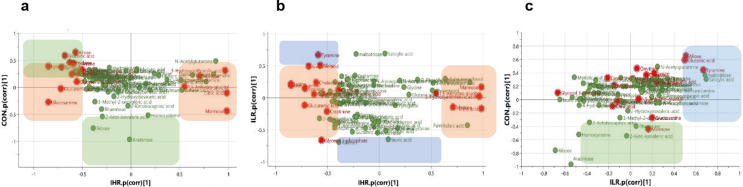
The SUS-plot of the OPLS-DA models featuring correlation of metabolites. Comparison of metabolites between the CON and the IHR group (**a**), the ILR group and the IHR group (**b**), and the CON and the ILR group (**c**). SUS-plot, The Shared and unique structures plot; OPLS-DA, Orthogonal partial least squares-discriminant analysis; CON, control; ILR, informed-low risk; IHR, informed-high risk

Subsequently, the SUS-plot was conducted to identify metabolites that contributed to the difference between the two OPLS-DA models. A total of 19 unique metabolites were identified across the three SUS-plots were identified (depicted red color) (Fig. 3). In the IHR group at 3 months, 17 metabolites (allose, carnitine, cholesterol, citric acid, creatinine, gluconic acid, glucosamine, glucuronic acid, glycerol-3-phosphate, lysine, maltose, mannose, tyramine, tyrosine, 1,5-anhydro-glycitol, 3-hydroxyanthranilic acid, 4-hydroxyphenylacetic acid) were distinguishable from the CON group, while 18 metabolites (allose, cholesterol, citric acid, creatinine, cysteine, gluconic acid, glucosamine, glucuronic acid, glycerol-3-phosphate, lysine, maltose, mannose, ornithine, tyrosine, tyramine, 1,5-anhydro-glycitol, 3-hydroxyanthranilic acid, 4-hydroxyphenylacetic acid) differed from the ILR group.

The ANOVA test for cross-validation indicated a significant decrease in the relative concentration of gluconic acid in the IHR group at 6 months compared to the baseline, whereas there were no significant changes in the CON and ILR groups. There were no significant differences in the magnitude of change of the relative levels of cholesterol among groups. However, the relative levels of the blood cholesterol were significantly lower in the IHR group at 3 months than that in the CON group (*p* = 0.036). In addition, the IHR group showed a significant decrease in the relative levels of gluconic acid at both 3 and 6 months compared to the baseline, as well as a significant decrease in the relative levels of lysine at 6 months compared to the baseline. A corresponding decrease was also observed in the CON group, which exhibited greater changes in gluconic acid levels that the ILR group at 3 months, along with a significant decrease in lysine levels at 6 months relative to the baseline (Supplementary Table 6).

### The impact of PA and dietary intake on blood cholesterol

In the IHR group, a negative relationship was observed between the relative levels of serum cholesterol and moderate PA (MPA, MET-hr/week) at 3 months. For every 1 MET-hr/week increase in MPA, serum cholesterol decreased by 3.5 (standardized β= -0.386, *p* < 0.05) after adjusting confounding factors including sex, education level, age, weight, energy intake, and fat intake. However, no causal relationship was found between cholesterol and MPA in the CON group (Table 3).

## Discussion

In this study, we aimed to investigate the impact of the disclosure of genetic information related to increased risk of weight gain and high BMI on changes in health-related behaviors such as dietary intake and PA. Individuals who perceived a higher genetic risk for BMI showed an increase in PA as well as an increased intake of dietary fat, fast food, and SSBs. Additionally, among those who recognized a higher genetic risk, an increase in PA was observed to be associated with a decrease in serum cholesterol levels. Several studies have primarily focused on changes in dietary intake following the recognition of genetic information [22, 41, 42]. However, most of these studies have provided participants with genetic information related to disease risk, and there is a lack of research exploring the impact of genetic information recognition on wellness-related areas. Genetic variations in *FTO*, *BDNF*, and *MC4R* genes, related to increased risk of weight gain, have been reported to be linked to elevated BMI and obesity risk in large-scale genome-wide association studies [7–9, 11]. BMI is acknowledged as a modifiable factor that can be influenced by lifestyle factors, including dietary intake and PA [20]. Although BMI is a widely utilized indicator of adiposity in public health, it has limitations, including its inability to differentiate between fat and lean mass and its limited capacity to accurately represent metabolic health [43, 44]. Additional measures, such as waist circumference or body composition analysis, may provide a more comprehensive assessment of obesity [45, 46]. Despite these limitations, BMI remains a practical and widely employed indicator in genetic studies of obesity [47, 48]. In the present study, genetic information linked to an increased risk of weight gain was provided with the expectation that it could encourage positive changes in health behaviors and reduce disease risk. There were no significant differences in BMI at the baseline among the CLR, CHR, ILR, and IHR groups. However, when participants were stratified by genetic risk score, the high-risk group (GS: 2.4–6.1) showed a significantly higher BMI compared to the low-risk group (GS: 1–2.3). This result is consistent with findings from previous genome-wide association studies reporting that individuals with higher polygenic risk for obesity showed positive association with increased BMI and a greater susceptibility to weight gain [49, 50]. Despite this difference, BMI values for both groups were within the normal range. Similarly, although the high-risk group was slightly older on average (low-risk: 27.4 ± 1.9 years; high-risk: 28.7 ± 2.2 years), both groups fell within the their late twenties, suggesting limited influence on the overall study outcomes. However, both age and BMI were adjusted for in the regression analysis, thereby minimizing potential confounding effects.

In this study, participants in the IHR group showed a significant increase in dietary fat and SSB consumption at 3 months. In contrast, the CON group exhibited a significant reduction in SSB intake at both 3 and 6 months, along with increased PA levels at 6 months compared to the ILR group. Since the information in genetic test result was not disclosed to the CON group, it can be speculated that the absence of genetic risk knowledge may have promoted positive behavior changes to mitigate potential health risks. Conversely, the increase in SSB consumption in the IHR group compared to the CON group suggest that the disclosure of genetic risk alone may not have been sufficient to promote favorable dietary changes. Moreover, participants’ fat intake (% energy/day) at baseline already exceeded the recommended range for Koreans (15–30%) according to the 2020 Dietary Reference Intakes for Koreans [38]. Therefore, the additional increase in fat intake observed in the IHR group may be considered an undesirable dietary change. While previous research has examined the impact of genetic risk recognition on dietary intake, findings remain inconclusive. A systematic literature review has reported that there were no significant changes in dietary intake or motivation following genetic risk perception [21]. In contrast, a randomized study conducted on 107 healthy individuals showed a significant improvement in dietary fat quality compared to a control group receiving general dietary recommendations [23]. Another study with 103 participants reported a significant reduction in salt intake when individuals were received advice based on genetic risk for the ACE gene [24]. However, these studies differ in design and methodology from the present study, suggesting the need for further clinical studies with consistent methodology to validate the results.

In this study, participants who were aware of genetic risk for BMI demonstrated a significant increase in MVPA and MPA at the 3-month follow-up compared to those who were unaware of genetic risk. Previous research examining the impact of the awareness of genetic risk for *FTO* rs9939609 reported no significant changes in PA levels during a 6-month intervention [27]. In contrast, another study found that individuals receiving personalized advice, which included genetic information related to BMI, exhibited significant improvements in PA after 6 months compared to those receiving general lifestyle advice without genetic information [28]. However, these studies provided counseling that could practically influence health behavior changes, distinguishing them from the present study, which solely provided genetic test results.

PA has been known to have a substantial impact on metabolic responses, leading to significant changes in the body’s homeostasis [51]. In this study, the intervention in the IHR group appeared to influence the metabolic profiles of the participants, particularly in relation to serum cholesterol levels. The regression analysis showed that for every 1 MET-hour per week increase in moderate MPA, serum cholesterol levels decreased by 3.5 at 3 months, indicating that increased PA contributed to a reduction in cholesterol concentrations. This finding aligns with previous studies linking PA to improvements in blood lipid profiles, including reductions in total cholesterol [52, 53]. Furthermore, both the IHR and the CON group showed a comparable change in the relative levels of gluconic acid and lysine at 6 months. This change can be linked to the increased MVPA levels in both groups in that increased PA enhances oxidative metabolism and the production of reactive oxygen species [54], leading to a reduction in plasma gluconic acid levels, which are consumed during antioxidant responses to oxidative stress [55]. Additionally, the decrease in serum lysine levels may be attributed to its mobilization during increased PA to support energy demands, which consequently leads to reduced plasma concentrations [56].

The mechanisms through which PA influences the lipid profile are not fully understood, but it is known that exercise enhances the ability of muscles to utilize lipid, rather than glycogen, leading to reduced blood lipid levels [57]. Additionally, exercise increases the activity of lecithin-cholesterol acyltransferase (LCAT), an enzyme involved in the esterification of cholesterol in the plasma, which facilitated the transfer of esters to HDL cholesterol [58]. Furthermore, increased PA has been reported to induce lipoprotein lipase activity, although the results have been inconsistent [59]. The process known as “cholesterol reverse transport”, which involves the removal of cholesterol from the body, increases after both acute and chronic exercise. This process is characterized by an increase in LCAT activity and a decrease in cholesteryl ester transfer protein (CETP), ultimately facilitating the clearance of circulating cholesterol [60].

This study has the advantage of evaluating the impact of recognizing genetic information associated with BMI on both PA and dietary intake, as well as blood metabolite levels, thereby confirming the physiological changes related to behavioral changes. Additionally, because the genetic information provided in this study was focused on wellness rather than disease, it allowed for the observation of changes in health-related behaviors following the recognition of genetic information from a preventive perspective before the onset of diseases. However, generalizing the results of the study may be challenging, as the participants were healthy individuals without any pre-existing conditions. Although overweight individuals were included as study participants, applying the findings to those with obesity, metabolic disorders, and other chronic conditions may be impractical. Furthermore, the blood metabolites measured in this study reflected dietary intake and PA over the past week, rather than being assessed immediately after meals and exercise, which represents a limitation of this study. In addition, this study focused exclusively on small-molecule metabolites measured by GC-MS/MS, without including the analysis of larger and more complex molecules such as lipid species. Future research should consider including a broader lipid profile analysis to gain a more comprehensive understanding of metabolic changes. Lastly, detailed socioeconomic information was not collected in this study. Given the potential influence of socioeconomic factors on health behaviors, future research should include this data to better account for confounders when interpreting the results.

In conclusion, this study demonstrated that disclosure of BMI-related genetic risk led to increased PA but did not improve dietary behaviors, with increased intake of dietary fat and SSBs. Furthermore, the results emphasized the important role of PA in improving cholesterol levels among individuals with high genetic risk. However, those who were not informed of their genetic results also exhibited favorable changes in PA, suggesting that the absence of genetic risk information may have encouraged positive behavioral changes. These findings suggest that genetic risk disclosure alone is insufficient to drive comprehensive behavior change. Therefore, a more holistic approach is required integrating genetic risk information with personalized counseling and continuous education to effectively support lifestyle modification.

**Table 1:** Baseline characteristics of the study participants ^1^

	All participants (*n* = 100)	CLR (*n* = 18)	CHR (*n* = 17)	ILR (*n* = 29)	IHR (*n* = 36)	*P* value ^1^
Sex n (%)						0.543
Men	50 (50.0)	8 (44.4)	9 (52.9)	12 (41.4)	21 (58.3)	
Women	50 (50.0)	10 (55.6)	8 (47.1)	17 (58.6)	15 (41.7)	
Education (%)						0.107
Undergraduate	6 (6.0)	0 (0.0)	2 (11.1)	4 (12.5)	0 (0.0)	
Bachelor’s degree	39 (39.0)	10 (58.8)	4 (22.2)	12 (37.5)	13 (39.4)	
Master’s degree	55 (55.0)	7 (41.2)	12 (66.7)	16 (50.0)	20 (60.6)	
Mean (SD)						
Age (years)	28.1 (2.1)	27.6 (1.8) ^ab^	28.3 (1.5) ^ab^	27.3 (1.9) ^b^	28.9 (2.5) ^a^	0.025
BMI (kg/m^2^)	22.3 (2.0)	22.1 (2.7) ^ab^	22.7 (1.5) ^ab^	21.3 (1.9) ^b^	23.0 (1.9) ^a^	0.008
Body fat mass (kg)						
Men	15.5 (4.0)	15.9 (1.4)	16.2 (1.3)	13.7 (1.2)	16.0 (0.9)	0.388
Women	15.8 (3.2)	15.2 (0.9) ^ab^	18.0 (0.9) ^a^	14.3 (0.7) ^b^	16.7 (0.9) ^ab^	0.026
Skeletal muscle mass (kg)						
Men	32.1 (3.7)	32.7 (1.7)	30.6 (1.4)	31.0 (1.1)	33.2 (0.6)	0.224
Women	21.2 (1.9)	21.9 (0.5)	21.2 (0.7)	20.9 (0.5)	21.2 (0.4)	0.677
Total PA (MET-hrs/week)	34.3 (22.7)	32.8 (21.0)	30.9 (20.3)	36.8 (26.9)	34.6 (21.3)	0.942
Energy (kcal/d)						
Men	2106.3 (65.7)	2129.2 (136.0)	2182.9 (87.7)	2096.8 (158.5)	2070.2 (115.2)	0.945
Women	1670.9 (58.5)	1644.6 (155.5)	1824.1 (103.9)	1685.2 (105.2)	1590.4 (104.6)	0.644
Carbohydrate (% energy/d)	47.8 (8.0)	51.3 (6.7)	47.1 (6.1)	46.6 (8.6)	47.4 (8.7)	0.129
Protein (% energy/d)	16.3 (3.7)	15.1 (3.4)	16.5 (3.1)	16.4 (4.6)	16.8 (3.3)	0.087
Fat (% energy/d)	32.5 (5.9)	31.1 (6.2)	33.9 (4.5)	33.0 (6.4)	32.1 (5.9)	0.508

^**1**^ P value was determined by using one-way ANOVA and Kruskal-Wallis tests. Means with different superscripts indicate the significant differences in changes in baseline characteristics among the groups, assessed by Bonferroni-correction for multiple comparisons

BMI, body mass index; PA, physical activity; MET: metabolic equivalent task; CLR, control-low risk; CHR, control-high risk; ILR, informed-low risk; IHR, informed high risk

**Table 2:** Comparison of the intakes of macronutrients and foods among the CON, ILR, and IHR groups ^1, 2, 3^

Characteristics	CON (*n* = 35)		ILR (*n* = 29)		IHR (*n* = 36)			*P* value^2^	
	Mean (SEM)	Change ^1^	Mean (SEM)	Change ^1^	Mean (SEM)	Change ^1^			
Energy (kcal/d)									
Men									
Baseline	2157.6 (76.7)		2096.8 (158.5)		2070.2 (115.2)				
3-month f/u	1877.2 (93.6) ^*^	-280.5 (73.3)	1745.0 (154.2)	-351.8 (228.4)	1894.7 (97.1)	-175.5 (110.8)	0.620		
6-month f/u	1945.9 (79.2) ^*^	-211.7 (66.9)	1618.1(141.2)^*^	-478.7 (201.7)	2094.9 (81.2)	24.8 (143.7)	0.119		
Women									
Baseline	1724.4 (97.8)		1685.2 (105.2)		1590.4(104.6)				
3-month f/u	1518.8 (71.5)	-205.5 (118.9)	1595.6 (110.4)	-72.9 (149.2)	1517.6(122.9)	-72.9 (149.2)	0.442		
6-month f/u	1673.7 (96.2)	-50.7 (122.3)	1466.8 (77.0) ^*^	-218.4 (93.3)	1570.0(145.3)	-20.4 (184.4)	0.462		
Carbohydrate (% energy/d)									
Baseline	49.2 (1.1)		46.6 (1.6)		47.4 (1.4)				
3-month f/u	54.9 (1.8) ^*^	5.6 (2.0)	46.2 (1.6)	-0.4 (2.1)	50.4 (2.5)	2.9 (2.7)	0.078		
6-month f/u	48.8 (1.4)	-0.5 (1.4)	50.1 (1.6)	3.5 (1.7)	48.0 (1.7)	0.5 (1.9)	0.259		
Protein (% energy/d)									
Baseline	15.8 (0.5)		16.4 (0.9)		16.8 (0.6)				
3-month f/u	16.4 (0.7)	0.7 (0.7)	18.1 (1.0)	1.8 (1.1)	18.0 (0.8)	1.2 (0.9)	0.783		
6-month f/u	16.8 (0.5)	1.1 (0.6)	16.2 (0.5)	-0.2 (1.0)	17.2 (0.6)	0.4 (0.8)	0.542		
Fat (% energy/d)									
Baseline	32.5 (0.9)		33.0 (1.2)		32.1 (1.0)				
3-month f/u	31.8 (1.6)	-0.8 (1.5)	34.4 (1.3)	1.4 (1.5)	35.6 (1.8)	3.5 (1.9)	0.059		
6-month f/u	33.2 (1.1)	0.7 (1.0)	32.5 (1.3)	-0.5 (1.5)	33.3 (1.5)	1.2 (1.7)	0.707		
Sugar-sweetened beverages (g/d)									
Baseline	87.6 (20.3)		52.1 (16.0)		28.6 (11.0)				
3-month f/u	36.9 (11.0) ^*^	-50.8 (24.0) ^b^	42.0 (11.7)	-10.1 (19.9) ^ab^	53.4 (19.5)	24.8 (22.2) ^a^	0.027		
6-month f/u	44.8 (15.9) ^*^	-42.8 (21.3) ^b^	54.9 (15.6)	2.8 (19.6) ^ab^	76.9 (20.4) ^*^	48.2 (18.0) ^a^	0.004		
Fast foods (g/d)									
Baseline	67.0 (14.1)		69.5 (19.8)		34.8 (9.2)				
3-month f/u	49.4 (12.0)	-17.7 (14.7)	67.0 (15.0)	-2.5 (26.1)	58.8 (15.6)	24.1 (17.2)	0.181		
6-month f/u	54.4 (14.5)	-12.7 (15.7)	46.8 (9.1)	-22.7 (21.0)	66.1 (15.3)	31.3 (17.0)	0.065		

^1^ Change = measurements at follow-up time-point– measurements at baseline

^2^ P value was calculated using one-way ANOVA and Kruskal-Wallis tests followed by Bonferroni-correction for multiple comparisons to determine the differences in the changes in dietary intakes among the CON, ILR, and IHR groups

^3^ The asterisk indicates significant differences (*P < 0.05*) in the dietary intakes from the baseline to the follow-up time point

CON, control (unaware); ILR, informed-low risk; IHR, informed-high risk

**Table 3:** Association between the MPA and the relative cholesterol level at 3-month follow-up

		IHR group				
		Cholesterol level at 3-month				
		B (SEM)	95% CI	Standardized β	t	*P value*
Model 1	MPA (MET-hrs/week)	-0.59 (1.52)	-3.678, 2.494	-0.067	-0.390	0.699
Model 2	Sex	28.5 (77.8)	-130.8, 187.8	0.120	0.366	0.717
	Education level	-24.5 (38.5)	-103.3, 54.3	-0.113	-0.637	0.529
	Age (years)	-12.2 (9.1)	-30.9, 6.4	-0.252	-1.343	0.190
	Weight (kg)	5.0 (3.5)	-2.2, 12.1	0.464	1.415	0.168
	Energy intake (kcal/d)	-0.1 (0.1)	-0.235, -0.031	-0.547	-2.662	0.013
	Fat intake (% energy/d)	-5.4 (2.0)	-9.5, -1.3	-0.501	-2.705	0.011
	MPA (MET-hrs/week)	-3.4 (1.7)	-6.8, -0.04	-0.386	-2.071	0.048

Estimates are presented relative to the reference group for categorical variables and per unit increment for continuous variables; sex (reference = men); education level (reference = undergraduate); age in 1-year increment; weight in 1 kg increment; energy intake in 1 kcal/d; fat intake in 1% energy/d; MPA in 1 MET-hrs/week increment

MPA, moderate physical activity; IHR, informed-high risk

## Electronic supplementary material

Below is the link to the electronic supplementary material.

Supplementary Material 1

## Abbreviations

BMI: Body mass index
MVPA: Moderate to vigorous physical activity
MPA: Moderate physical activity
PA: Physical activity
SSB: Sugar-sweetened beverage
VPA: Vigorous physical activity
