# Supplementary material for: Impact of recognition of genetic information related to BMI on changes in physical activity, dietary intake, and blood cholesterol level: a randomized controlled trial
Source: Eur J Nutr. 2025 May 27;64(5):190. doi: 10.1007/s00394-025-03713-x (PMC12116609; doi:10.1007/s00394-025-03713-x)
Supplement: Supplementary file 1 — Supplementary Material 1 [file 394_2025_3713_MOESM1_ESM.docx]

- Article title: Impact of recognition of genetic information related to BMI on changes in physical activity, dietary intake, and blood cholesterol level: A randomized controlled trial

- Journal name: European Journal of nutrition

- Author names: Ga Young Lee ^1^, Junghak Lee ^2^, Jeong-Han Kim ^2^, Kyong-Mee Chung^3^, Sung Nim Han ^1, 4, *^

^1^ Department of Food and Nutrition, College of Human Ecology, Seoul National University, Seoul, Republic of Korea

^2^ Department of Agricultural Biotechnology and Research Institute of Agriculture and Life Sciences, Seoul National University, Seoul, Republic of Korea

^3^ Department of Psychology, Yonsei University, Seoul, Korea

^4^ Research Institute of Human Ecology, Seoul National University, Seoul, Republic of Korea

Gayoung Lee, [lgykiki90@snu.ac.kr](mailto:lgykiki90@snu.ac.kr); Junghak Lee, [crane245@snu.ac.kr](mailto:crane245@snu.ac.kr); Jeong-Han Kim, [kjh2404@snu.ac.kr](mailto:kjh2404@snu.ac.kr); Kyong-Mee Chung, [kmchung@yonsei.ac.kr](mailto:kmchung@yonsei.ac.kr)

*** Correspondence:**Dr. Sung Nim Han, [snhan@snu.ac.kr](mailto:snhan@snu.ac.kr),

Department of Food and Nutrition, College of Human Ecology, Seoul National University

1 Gwanak-ro, Gwanak-gu, Seoul, 08826, Korea. Phone: +82-2-880-6836

**Supplementary Table 1. Genetic test result for BMI related genes**

| **Test Item** | **Gene** | **SNP** | **Overall results** | **Descriptions on possessing risk allele** | **Recommendation for possessing risk allele** |
| --- | --- | --- | --- | --- | --- |
| **Body Fat Mass** | FTO | rs9939609 | Good / Borderline risk / Caution | There is a high risk of obesity by excessively storing the remaining calories in fat. | Low-fat meals are recommended. |
|  | MC4R | rs17782313 |  | There is a high risk of obesity by increasing appetite and eating snacks frequently. | It is recommended to reduce snack intake. |
|  | BDNF | rs6265 |  | Social stress can increase appetite as a reward. | Be careful of overeating or binge eating even in stressful or depressive situations. |

**Supplementary Table 2. International physical activity questionnaire (IPAQ)-short form**

| **International physical activity questionnaire (IPAQ)-short form** | |
| --- | --- |
| **The questions will ask you about the time you spent being physically active in the last 7 days. Please answer each question even if you do not consider yourself to be an active person. Please think about the activities you do at work, as part of your house and yard work, to get from place to place, and in your spare time for recreation, exercise or sport.** | |
| Think about all the vigorous activities that you did in the last 7 days. Vigorous physical activities refer to activities that take hard physical effort and make you breathe much harder than normal. Think only about those physical activities that you did for at least 10 minutes at a time. | |
| 1. | During the last 7 days, on how many days did you do vigorous physical activities like heavy lifting, digging, aerobics, or fast bicycling? |
|  | _____ days per week   - No vigorous physical activities → Skip to question 3 |
| 2. | How much time did you usually spend doing vigorous physical activities on one of those days? |
|  | _____ hours per day _____ minutes per day   - Don’t know/Not sure |
| Think about all the moderate activities that you did in the last 7 days. Moderate activities refer to activities that take moderate physical effort and make you breathe somewhat harder than normal. Think only about those physical activities that you did for at least 10 minutes at a time. | |
| 3. | During the last 7 days, on how many days did you do moderate physical  activities like carrying light loads, bicycling at a regular pace, or doubles tennis? Do not include walking. |
|  | _____ days per week   - No moderate physical activities → Skip to question 5 |
| 4. | How much time did you usually spend doing moderate physical activities on one of those days? |
|  | _____ hours per day _____ minutes per day  Don’t know/Not sure |
| Think about the time you spent walking in the last 7 days. This includes at work and at home, walking to travel from place to place, and any other walking that you have done solely for recreation, sport, exercise, or leisure. | |
| 5. | During the last 7 days, on how many days did you walk for at least 10 minutes  at a time? |
|  | _____ days per week   - No walking → Skip to question 7 |
| 6. | How much time did you usually spend walking on one of those days? |
|  | _____ hours per day  _____ minutes per day  Don’t know/Not sure |
| The last question is about the time you spent sitting on weekdays during the last 7 days. Include time spent at work, at home, while doing course work and during leisure time. This may include time spent sitting at a desk, visiting friends, reading, or sitting or lying down to watch television. | |
| 7. | During the last 7 days, how much time did you spend sitting on a week day? |
|  | _____ hours per day _____ minutes per day   - Don’t know/Not sure |

**Supplementary Table 3. Baseline comparison of the BMI, dietary intake, and PA between the subjects with low risk and high risk of polygenic risk for *FTO*, *MC4R*, and *BDNF* genes ^1,2^**

| **Baseline Characteristics** | **Subjects at Low risk**  **(GS=0-2.3, n = 47)** | **Subjects at High risk**  **(GS=2.4-6.1, n = 53)** | ***P* value** |
| --- | --- | --- | --- |
| **Age (years)** | 27.4 (1.9) | 28.7 (2.2) | 0.003 |
| **BMI (kg/m^2^)** | 21.6 (2.1) | 22.9 (1.8) | 0.001 |
| **Total PA (MET-hrs/week)** | 35.3 (24.7) | 33.4 (20.9) | 0.682 |
| **Energy (kcal/d)** | 1857.2 (505.9) | 1916.4 (476.9) | 0.549 |
| **Carbohydrate (% energy/d)** | 48.4 (8.2) | 47.3 (7.9) | 0.495 |
| **Protein (% energy/d)** | 15.9 (4.2) | 16.7 (3.2) | 0.273 |
| **Fat (% energy/d)** | 32.3 (6.3) | 32.7 (5.5) | 0.721 |

^1^ The data are presented as mean (SEM).

^2^ P value was calculated using independent t-test and Wilcoxson’s Rank sum test.

GS, genetic score; BMI, body mass index; PA, physical activity; MET: metabolic equivalent task

**Supplementary Table 4.** **Comparison of the anthropometric measurements** **among the CON, ILR, and IHR groups ^1, 2, 3^**

| **Characteristics** | **CON**  **(n = 17)** | | **ILR**  **(n = 12)** | | **IHR**  **(n = 21)** | | ***P***  **value ^2^** |
| --- | --- | --- | --- | --- | --- | --- | --- |
|  | **Mean (SEM)** | **Change ^1^** | **Mean (SEM)** | **Change ^1^** | **Mean (SEM)** | **Change ^1^** |  |
| **Men** | | | | | | | |
| **BMI (kg/m^2^)** | | | | | | | |
| **Baseline** | 23.8 (0.3) |  | 22.5 (0.4) |  | 24.0 (0.3) |  |  |
| **3-month f/u** | 23.7 (0.3) | -0.06 (0.13) | 22.4 (0.4) | -0.06 (0.15) | 23.8 (0.3) | -0.12 (0.11) | 0.697 |
| **6-month f/u** | 23.9 (0.4) | 0.10 (0.19) | 22.6 (0.4) | 0.12 (0.15) | 23.9 (0.3) | -0.03 (0.13) | 0.761 |
| **Weight (kg)** | | | | | | | |
| **Baseline** | 72.1 (1.9) |  | 68.9 (2.1) |  | 74.3 (1.3) |  |  |
| **3-month f/u** | 72.0 (2.0) | -0.2 (0.4) | 68.7 (1.8) | -0.2 (0.5) | 73.9 (1.3) | -0.4 (0.3) | 0.781 |
| **6-month f/u** | 72.5 (2.1) | 0.4 (0.6) | 69.2 (1.8) | 0.3 (0.4) | 74.1 (1.1) | -0.2 (0.4) | 0.695 |
| **Skeletal muscle mass (kg)** | | | | | | | |
| **Baseline** | 31.6 (1.1) |  | 31.0 (1.1) |  | 33.2 (0.6) |  |  |
| **3-month f/u** | 31.8 (1.2) | 0.2 (0.4) | 31.5 (0.9) | 0.5 (0.3) | 33.4 (0.7) | 0.2 (0.3) | 0.840 |
| **6-month f/u** | 31.9 (1.3) | 0.3 (0.4) | 31.2 (1.0) | 0.2 (0.3) | 33.4 (0.7) | 0.2 (0.3) | 0.541 |
| **Body fat mass (kg)** | | | | | | | |
| **Baseline** | 16.1 (0.9) |  | 13.7 (1.2) |  | 16.0 (0.9) |  |  |
| **3-month f/u** | 15.7 (0.9) | -0.4 (0.5) | 12.8 (1.3) **^*^** | -1.0 (0.4) | 14.9 (0.8) **^*^** | -1.2 (0.3) | 0.595 |
| **6-month f/u** | 15.9 (0.9) | -0.2 (0.6) | 13.7 (1.3) | -0.0 (0.4) | 15.2 (0.8) **^*^** | -0.8 (0.4) | 0.323 |
| **Women** | | | | | | | |
| **Characteristics** | **CON**  **(n = 18)** | | **ILR**  **(n = 17)** | | **IHR**  **(n = 15)** | | ***P***  **value ^2^** |
|  | **Mean (SEM)** | **Change ^1^** | **Mean (SEM)** | **Change ^1^** | **Mean (SEM)** | **Change ^1^** |  |
| **BMI (kg/m^2^)** | | | | | | | |
| **Baseline** | 21.1 (0.3) |  | 20.4 (0.4) |  | 21.6 (0.5) |  |  |
| **3-month f/u** | 20.9 (0.4) | -0.2 (0.2) | 20.1 (0.4) **^*^** | -0.4 (0.1) | 21.1 (0.5) | -0.4 (0.3) | 0.693 |
| **6-month f/u** | 20.6 (0.3) **^*^** | -0.4 (0.2) | 20.2 (0.4) | -0.3 (0.1) | 21.3 (0.5) | -0.3 (0.3) | 0.778 |
| **Weight (kg)** | | | | | | | |
| **Baseline** | 56.3 (1.0) |  | 53.0 (1.1) |  | 56.0 (1.5) |  |  |
| **3-month f/u** | 55.8 (1.1) | -0.5 (0.4) | 52.0 (1.1) **^*^** | -1.0 (0.3) | 54.9 (1.6) | -1.1 (0.7) | 0.714 |
| **6-month f/u** | 55.1 (0.8) **^*^** | -1.2 (0.5) | 52.3 (1.1) **^*^** | -0.7 (0.4) | 55.3 (1.6) | -0.7 (0.8) | 0.725 |
| **Skeletal muscle mass (kg)** | | | | | | | |
| **Baseline** | 21.6 (0.4) |  | 20.9 (0.5) |  | 21.2 (0.4) |  |  |
| **3-month f/u** | 21.9 (0.4) **^*^** | 0.3 (0.1) | 21.1 (0.5) | 0.2 (0.1) | 21.3 (0.5) | 0.1 (0.2) | 0.758 |
| **6-month f/u** | 21.7 (0.4) | 0.1 (0.2) | 21.0 (0.5) | 0.1 (0.1) | 21.3 (0.5) | 0.1 (0.2) | 0.929 |
| **Body fat mass (kg)** | | | | | | | |
| **Baseline** | 16.4 (0.7) |  | 14.3 (0.7) |  | 16.7 (0.9) |  |  |
| **3-month f/u** | 15.3 (0.7) **^*^** | -1.1 (0.4) | 13.0 (0.8) **^*^** | -1.3 (0.4) | 15.4 (1.0) **^*^** | -1.3 (0.5) | 0.936 |
| **6-month f/u** | 14.9 (0.6) **^*^** | -1.5 (0.5) | 13.4 (0.7) **^*^** | -0.9 (0.3) | 15.8 (1.0) | -0.9 (0.5) | 0.482 |

^1^ Change = measurements at follow-up time-point – measurements at baseline

^2^ P value was calculated using one-way ANOVA and Kruskal-Wallis tests to determine the differences in anthropometric measurement changes among the CON, ILR, and IHR groups.

^3^ The asterisk indicates significant differences (P<0.05) in the anthropometric measurements from the baseline to the follow-up time point.

**Supplementary Table 5.** **The intake of micronutrients among the CON, ILR, and IHR groups ^1, 2, 3, 4^**

| **Characteristics** | **CON**  **(n = 35)** | | **ILR**  **(n = 29)** | | **IHR**  **(n = 36)** | | ***P***  **value ^2^** |
| --- | --- | --- | --- | --- | --- | --- | --- |
|  | **Mean (SEM)** | **Change ^1^** | **Mean (SEM)** | **Change ^1^** | **Mean (SEM)** | **Change ^1^** |  |
| **Vitamin A (μg RAE/d)** | | | | | | | |
| **Baseline** | 198.9 (16.0) |  | 204.1 (10.9) |  | 219.6 (24.4) |  |  |
| **3-month f/u** | 229.6 (18.0) | 30.7 (23.3) | 249.9 (16.9) | 45.7 (18.8) | 271.5 (39.7) | 51.9 (34.6) | 0.996 |
| **6-month f/u** | 188.7 (11.5) | -10.2 (19.0) | 310.1 (71.7) | 105.9 (73.0) | 219.2 (21.9) | -0.4 (20.8) | 0.422 |
| **Vitamin D (μg/d)** | | | | | | | |
| **Baseline** | 2.1 (0.6) |  | 2.6 (0.5) |  | 2.2 (0.2) |  |  |
| **3-month f/u** | 2.0 (0.3) | -0.1 (0.7) | 4.1 (1.3) | 1.6 (1.4) | 1.7 (0.3) ^*^ | -0.5 (0.3) | 0.045 |
| **6-month f/u** | 1.9 (0.3) | -0.2 (0.7) | 2.4 (0.5) | -0.2 (0.7) | 2.7 (0.7) | 0.4 (0.7) | 0.795 |
| **Vitamin K (μg/d)** | | | | | | | |
| **Baseline** | 54.3 (5.7) |  | 54.3 (5.9) |  | 70.8 (12.2) |  |  |
| **3-month f/u** | 73.5 (8.2) | -19.1 (10.6) | 58.6 (10.5) | -4.3 (11.7) | 78.7 (12.9) | -7.9 (17.0) | 0.423 |
| **6-month f/u** | 59.1 (7.7) | -4.8 (9.6) | 61.5 (9.3) | -7.2 (11.6) | 45.6 (5.1) ^*^ | 25.2 (11.6) | 0.228 |
| **Vitamin C (mg/d)** | | | | | | | |
| **Baseline** | 35.8 (4.2) |  | 39.4 (6.9) |  | 57.8 (13.0) |  |  |
| **3-month f/u** | 28.7 (2.9) | 7.2 (5.3) | 33.6 (9.3) | 5.8 (11.7) | 32.2 (4.0) ^*^ | 25.6 (11.8) | 0.874 |
| **6-month f/u** | 26.9 (2.2) | 8.9 (5.1) | 25.6 (1.9) | 13.9 (6.7) | 29.5 (2.6) ^*^ | 28.3 (12.9) | 0.795 |
| **Sodium (mg/d)** | | | | | | | |
| **Baseline** | 1591.9 (60.6) |  | 1796.6 (93.3) |  | 1769.8 (88.2) |  |  |
| **3-month f/u** | 1894.4 (105.0) ^*^ | 302.5 (116.4) | 1912.3 (119.9) | 115.7 (138.5) | 1766.2 (108.7) | -3.6 (142.1) | 0.135 |
| **6-month f/u** | 1881.6 (103.1) ^*^ | 289.7 (105.7) | 2001.5 (84.4) | 204.9 (114.0) | 1923.8 (109.1) | 153.9 (144.2) | 0.947 |
| **Calcium (mg/d)** | | | | | | | |
| **Baseline** | 248.6 (14.2) |  | 253.7 (15.6) |  | 235.0 (19.2) |  |  |
| **3-month f/u** | 273.8 (20.2) | 25.2 (23.1) | 257.4 (15.9) | 3.7 (24.3) | 289.7 (28.0) | 54.6 (29.1) | 0.427 |
| **6-month f/u** | 252.2 (15.2) | 3.6 (16.0) | 227.8 (14.8) | -25.9 (21.0) | 241.6 (22.5) | 6.5 (21.5) | 0.499 |
| **Iron (mg/d)** | | | | | | | |
| **Baseline** | 7.0 (0.3) |  | 7.3 (0.4) |  | 8.6 (0.6) |  |  |
| **3-month f/u** | 8.7 (0.6) ^*^ | 1.7 (0.7) | 8.1 (0.5) | 0.8 (0.6) | 8.7 (0.6) | 0.1 (0.9) | 0.498 |
| **6-month f/u** | 8.3 (0.5) ^*^ | 1.3 (0.6) **^a^** | 8.0 (0.3) | 0.7 (0.5) **^ab^** | 7.9 (0.7) | -0.7 (1.0) **^b^** | 0.021 |
| **Fiber (g/d)** | | | | | | | |
| **Baseline** | 9.2 (0.4) |  | 9.7 (0.6) |  | 10.7 (0.9) |  |  |
| **3-month f/u** | 9.9 (0.5) | 0.7 (0.6) | 9.8 (0.7) | 0.1 (0.8) | 10.0 (0.8) | -0.7 (1.0) | 0.233 |
| **6-month f/u** | 9.4 (0.5) | 0.3 (0.6) | 9.9 (0.5) | 0.2 (0.7) | 8.6 (0.6) ^*^ | -2.1 (0.7) | 0.032 |
| **Folate (mg/d)** | | | | | | | |
| **Baseline** | 362.4 (18.3) |  | 356.8 (30.3) |  | 392.0 (22.2) |  |  |
| **3-month f/u** | 317.6 (18.9) | -0.0 (13.2) | 312.9 (22.6) | 0.9 (25.7) | 335.6 (17.5) | 21.7 (15.8) | 0.214 |
| **6-month f/u** | 321.4 (16.5) | 11.9 (12.2) **^ab^** | 286.9 (21.1) | 11.2 (18.9) **^b^** | 316.8 (15.4) ^*^ | 51.5 (14.4) **^a^** | 0.009 |
| **Saturated fatty acids (g)** | | | | | | | |
| **Baseline** | 10.8 (0.8) |  | 9.8 (0.9) |  | 12.8 (1.8) |  |  |
| **3-month f/u** | 10.5 (1.0) | -0.4 (1.2) | 9.6 (1.0) | -0.2 (1.0) | 11.3 (1.1) | -1.5 (1.9) | 0.811 |
| **6-month f/u** | 10.3 (0.9) | -0.5 (1.0) | 8.6 (0.8) | -1.2 (1.0) | 10.0 (1.1) | -2.7 (1.9) | 0.508 |
| **Monounsaturated fatty acids (g)** | | | | | | | |
| **Baseline** | 13.3 (1.0) |  | 11.2 (1.0) |  | 16.1 (2.4) |  |  |
| **3-month f/u** | 13.3 (1.3) | -0.1 (1.5) | 12.0 (1.3) | 0.8 (1.3) | 13.9 (1.2) | -2.2 (2.6) | 0.536 |
| **6-month f/u** | 12.7 (0.9) | -0.6 (1.3) | 10.6 (0.9) | -0.6 (1.2) | 12.4 (1.3) | -3.7 (2.6) | 0.395 |
| **Polyunsaturated fatty acids (g)** | | | | | | | |
| **Baseline** | 12.2 (1.0) |  | 10.7 (1.1) |  | 12.9 (1.3) |  |  |
| **3-month f/u** | 11.2 (1.0) | -1.0 (1.3) | 10.2 (1.0) | -0.6 (1.2) | 11.8 (1.0) | -1.1 (1.2) | 0.956 |
| **6-month f/u** | 11.7 (1.0) | -0.4 (1.3) | 9.7 (1.1) | -1.1 (1.4) | 11.2 (1.0) | -1..6 (1.3) | 0.813 |

^1^ Change = measurements at follow-up time-point – measurements at baseline

^2^ P value was calculated using one-way ANOVA and Kruskal-Wallis tests followed by Bonferroni tests for multiple comparisons to determine the differences in the changes in micronutrient intakes among the CON, ILR, and IHR groups.

^3^ The asterisk indicates significant differences (P<0.05) in the dietary intakes from the baseline to the follow-up time point.

^4^ Nutrient intakes were determined considering the usage of dietary supplements.

**Supplementary Table 6.** **Changes in the relative metabolite levels among the CON, ILR, and IHR groups ^1, 2, 3^**

|  | **CON (n=35)** | | **ILR (n=29)** | | **IHR (n=36)** | | ***P* value ^2^** |
| --- | --- | --- | --- | --- | --- | --- | --- |
|  | **Mean (SEM)** | **Change ^1^** | **Mean (SEM)** | **Change ^1^** | **Mean (SEM)** | **Change ^1^** |  |
| **Carbohydrates** | | | | | | | |
| **Gluconic acid** | | | | | | | |
| **Baseline** | 97.4 (74.7) |  | 7.9 (3.5) |  | 226.4 (97.1) |  |  |
| **3-month f/u** | 53.2 (33.2) | -44.2 (42.2) | 8.4 (4.6) | 0.6 (5.1) | 79.0 (36.8) ^*^ | -147.4 (86.2) | 0.111 |
| **6-month f/u** | 41.3 (24.3) | -56.1 (50.9) **^a^** | 8.0 (3.4) | 0.1 (3.5) **^a^** | 119.1 (66.8) ^*^ | -107.3 (51.8) **^b^** | 0.001 |
| **Glucuronic acid** | | | | | | | |
| **Baseline** | 11189.5 (936.2) |  | 11097.1 (609.4) |  | 13021.8 (1087.4) |  |  |
| **3-month f/u** | 10716.8 (858.4) | -472.7 (429.9) | 9775.7 (705.1) ^*^ | -1321.4 (529.7) | 12084.8 (955.0 ^*^ | -937.0 (629.5) | 0.776 |
| **6-month f/u** | 10727.8 (721.8) | -461.7 (492.8) | 10623.7 (619.6) | -473.4 (383.4) | 12469.3 (917.5) | -552.4 (671.4) | 0.809 |
| **Amino acids** | | | | | | | |
| **Cysteine** | | | | | | | |
| **Baseline** | 305227.3 (23281.8) |  | 327582.8 (21458.0) |  | 362166.3 (22368.3) |  |  |
| **3-month f/u** | 294967.6 (19095.4) | -10259.6 (19204.6) | 293566.0 (22406.5) | -34016.8 (16930.4) | 346077.9 (17604.7) | -16088.4 (18467.4) | 0.810 |
| **6-month f/u** | 310343.9 (22171.2) | 5116.7 (20069.3) | 304059.2 (21070.4) | -23523.6 (14287.4) | 343609.6 (18723.8) | -18556.7 (19333.3) | 0.236 |
| **Lysine** | | | | | | | |
| **Baseline** | 4851.2 (966.9) |  | 3485.1 (799.9) |  | 6298.4 (1084.3) |  |  |
| **3-month f/u** | 4384.1 (902.3) | -467.0 (213.3) | 3073.6 (734.5) | -411.5 (224.2) | 5715.8 (958.0) | -582.6 (403.1) | 0.609 |
| **6-month f/u** | 4243.8 (829.5) ^*^ | -607.3 (252.8) **^b^** | 3487.3 (796.6) | 2.2 (198.6) **^a^** | 5637.7 (937.4) ^*^ | -660.6 (395.6) **^b^** | 0.038 |
| **Organic acids** | | | | | | | |
| **Citric acid** | | | | | | | |
| **Baseline** | 19.0 (5.7) |  | 8.9 (3.4) |  | 18.0 (4.3) |  |  |
| **3-month f/u** | 14.3 (4.0) | -4.7 (6.1) | 18.9 (6.1) ^*^ | 10.0 (4.1) | 20.2 (5.8) | 2.2 (5.8) | 0.178 |
| **6-month f/u** | 18.5 (4.8) | -0.5 (5.7) | 14.6 (6.5) | 5.7 (5.0) | 27.4 (7.2) | 9.4 (5.8) | 0.973 |
| **Steroids** | | | | | | | |
| **Cholesterol** | | | | | | | |
| **Baseline** | 383.3 (90.7) |  | 297.5 (23.7) |  | 257.3 (33.7) |  |  |
| **3-month f/u** | 346.3 (40.1) | -37.0 (64.8) | 283.4 (26.3) | -14.1 (25.8) | 233.1 (19.8) | -24.3 (35.2) | 0.738 |
| **6-month f/u** | 284.4 (22.3) | -98.9 (84.8) | 264.1 (20.3) | -33.4 (23.6) | 239.6 (15.3) | -17.8 (35.5) | 0.424 |

^1^ Change = measurements at follow-up time-point – measurements at baseline

^2^ *P* value was calculated by using one-way ANOVA tests and Kruskal-Wallis tests to determine differences in the blood metabolite changes among the CON, ILR, and IHR groups.

^3^ The asterisks indicate significant difference (*P*<0.05) in the metabolites from baseline to 3-month follow-up. CON, control; ILR, informed-low risk; IHR, informed-high risk

**Supplementary F****igure 1.**

**
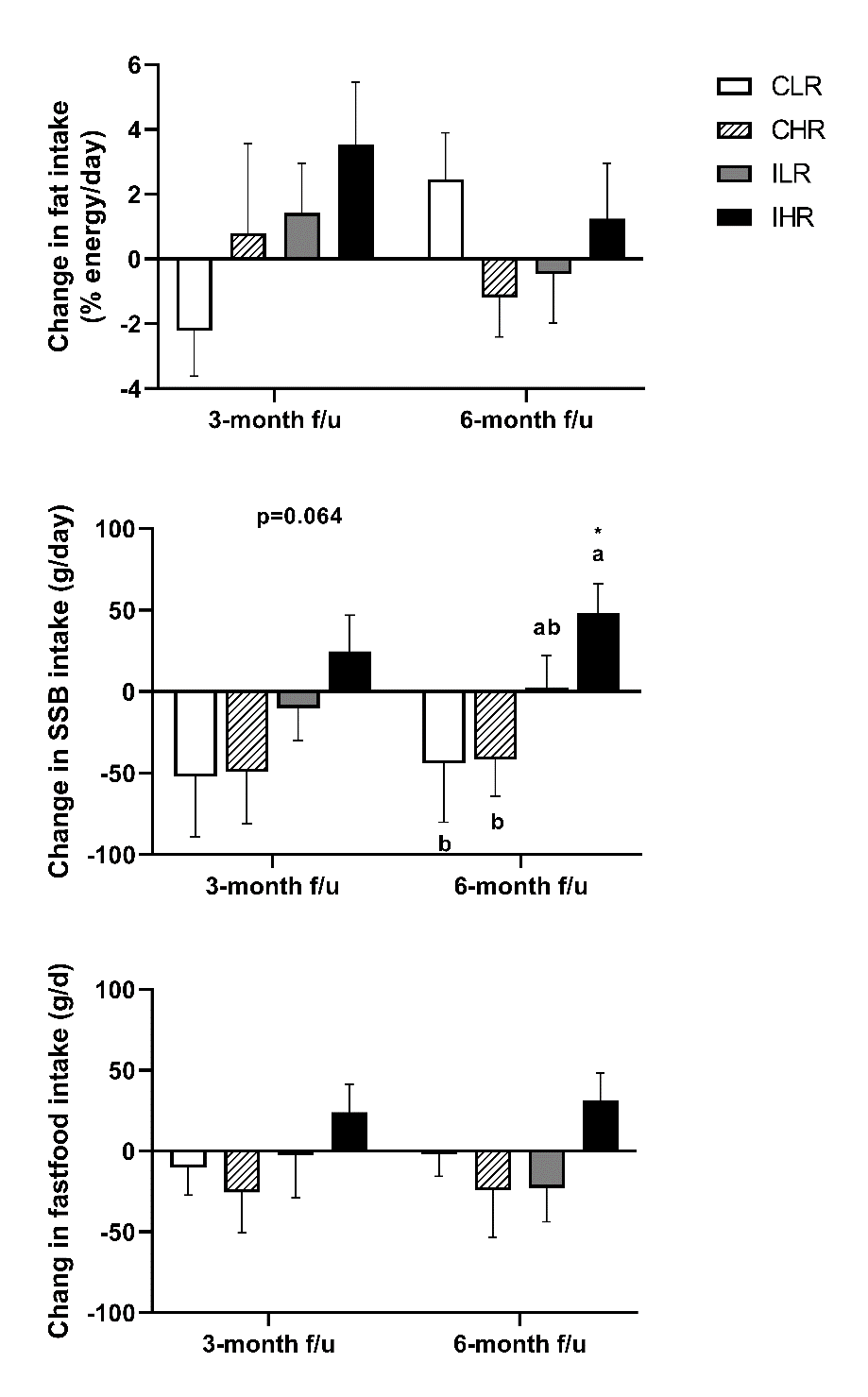
**

**Supplementary Figure 2. The PLS-DA score plot based on the GC-MS/MS spectra of the metabolites from the CON, ILR, and IHR groups**


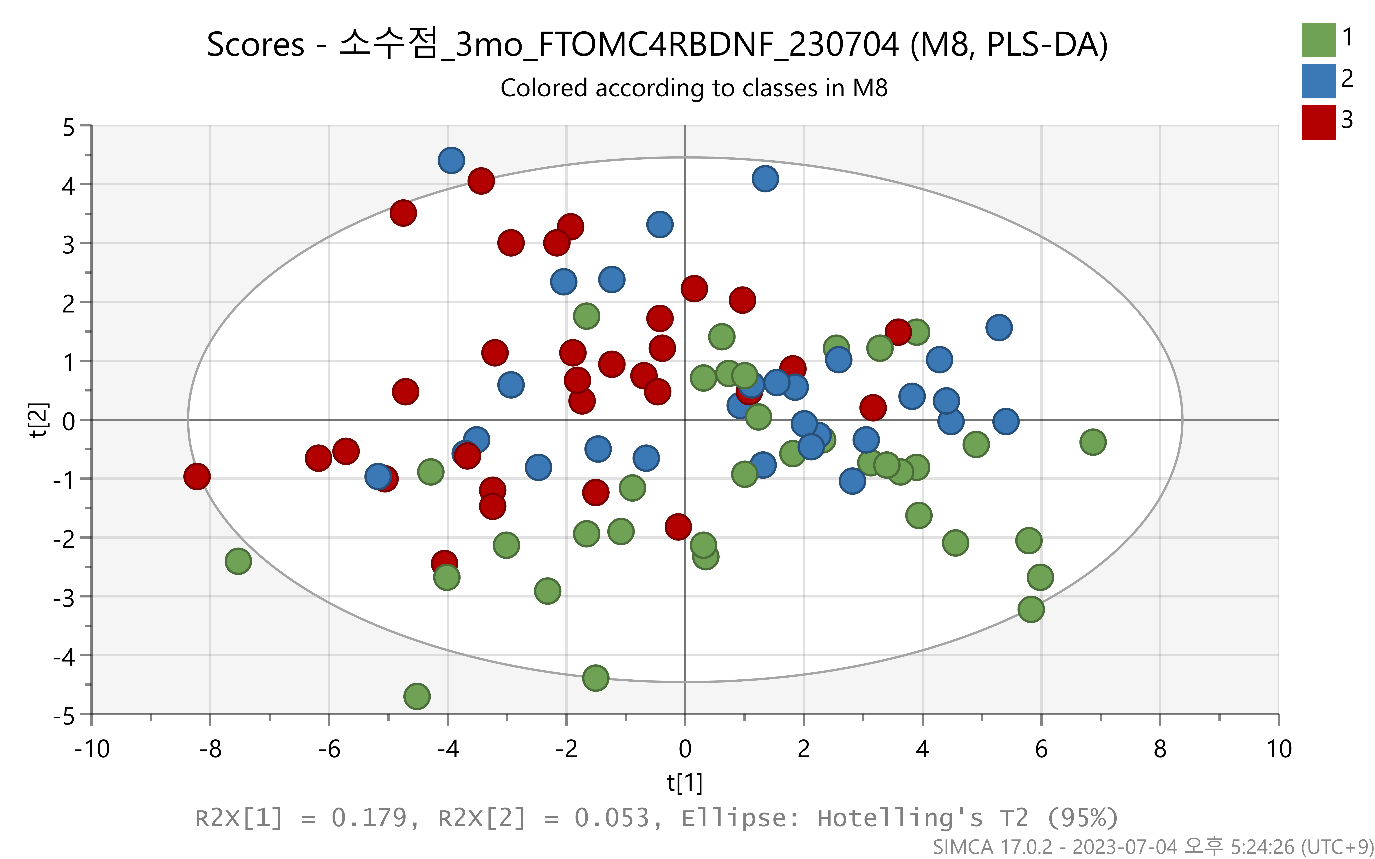


CON (marked green), n=35; ILR (marked blue), n=29; IHR (marked red), n=31

PLS-DA, partial least squares-discriminant analysis

**Supplementary Figure 3. The PCA score plot of metabolites of the CON, ILR, and IHR groups**


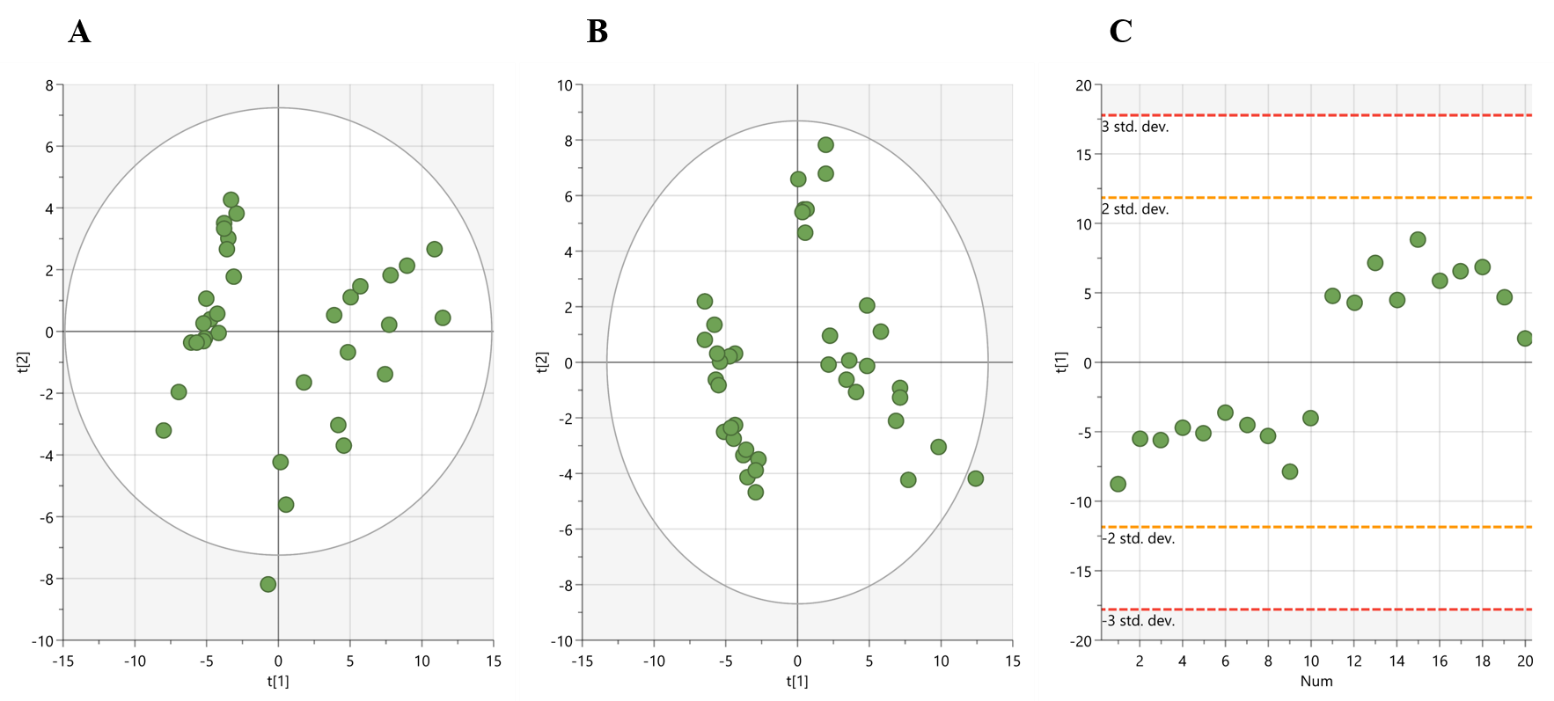

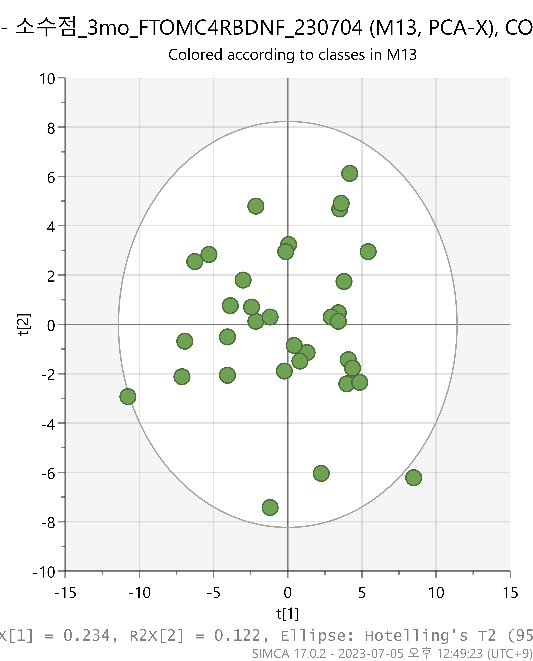

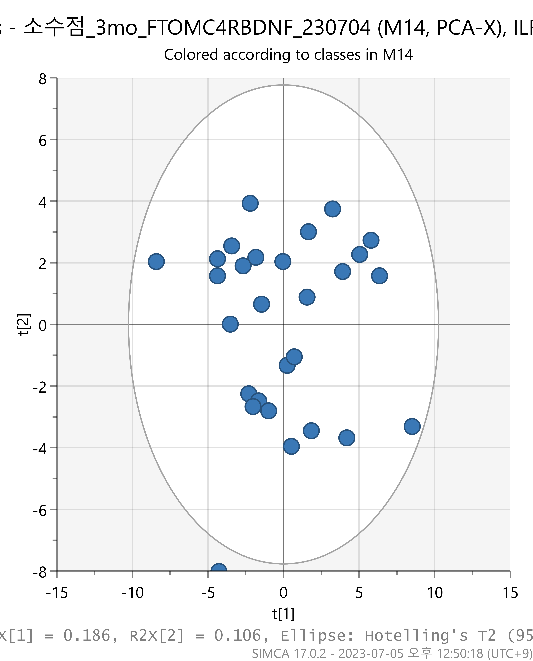

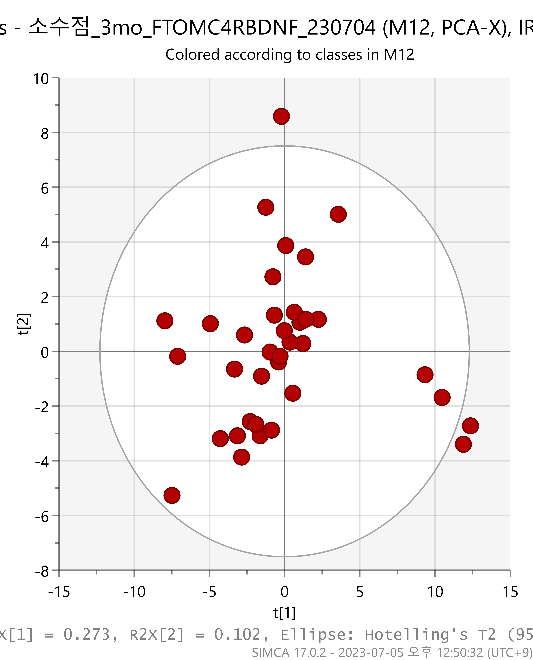


The fitness values of R2X and Q2 of the PCA models of each group are as follows; CON group (A): R2X=0.355, Q2=0.176; ILR group (B): R2X=0.292, Q2=0.076; IHR group (C): R2X=0.374, Q2=0.187.

PCA, principal component analysis; CON, control; ILR, informed-low risk; IHR, informed-high risk

**Supplementary Figure 4.** **The OPLS-DA score plots and the permutation test plots of the CON, ILR, and IHR groups**


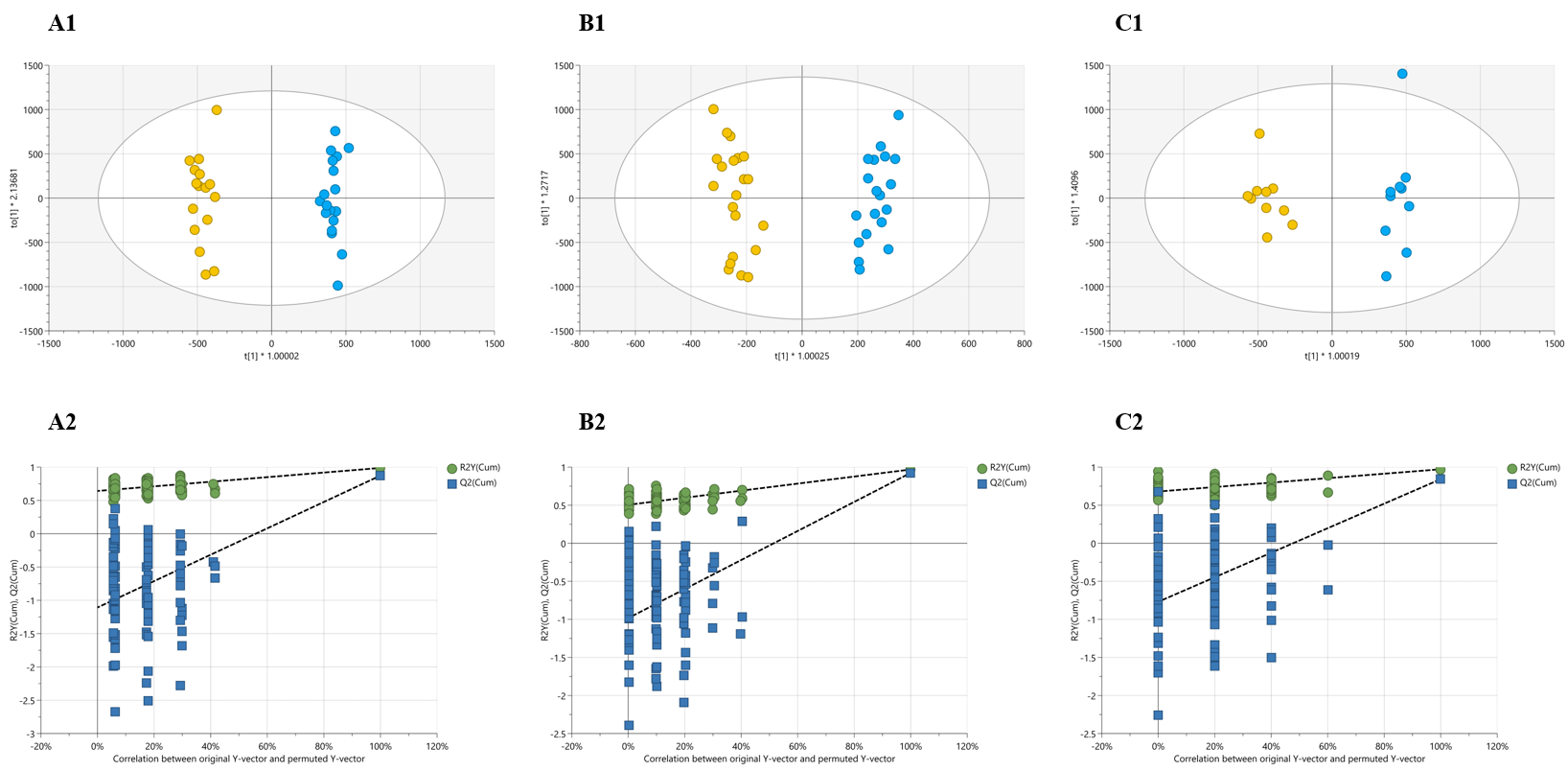

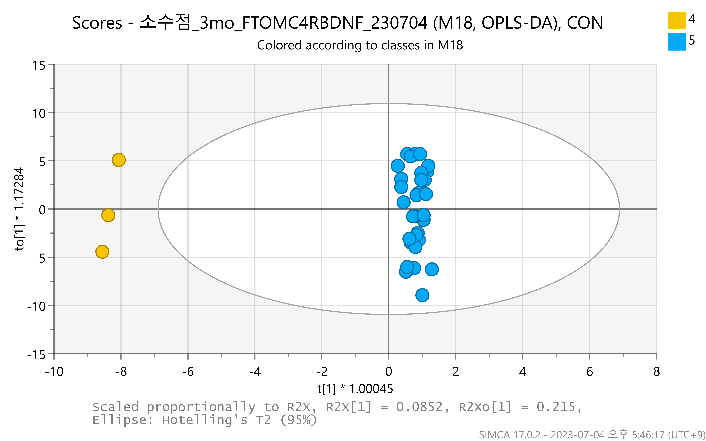

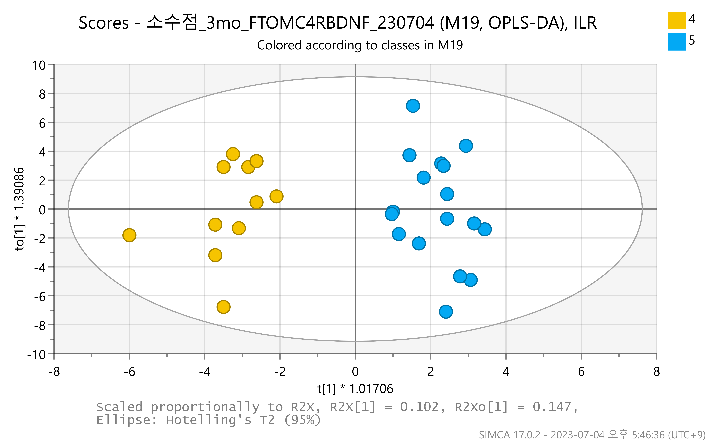

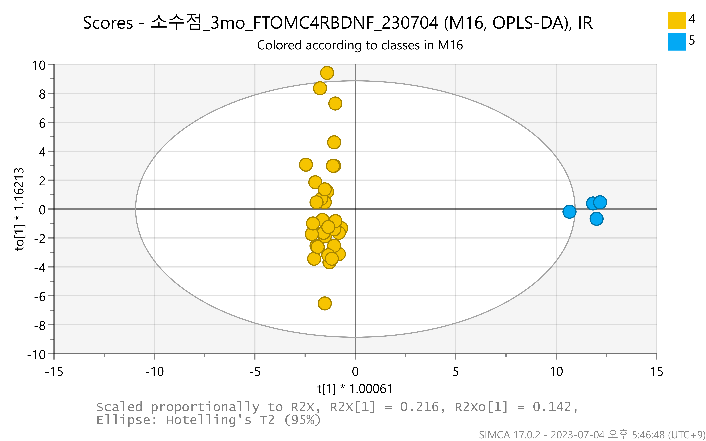

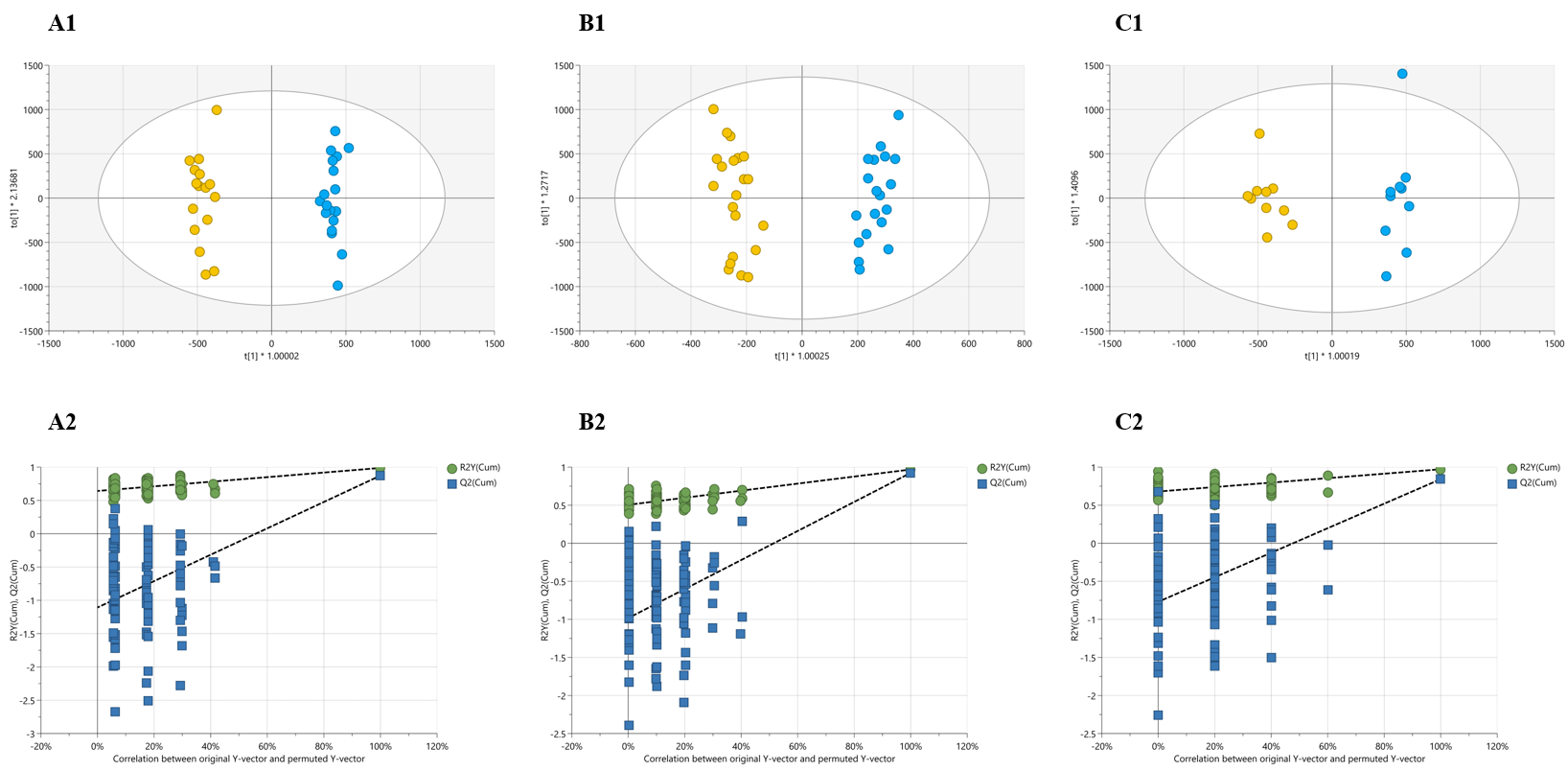

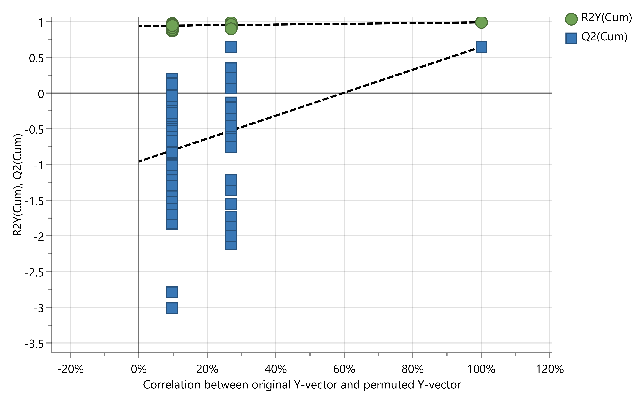

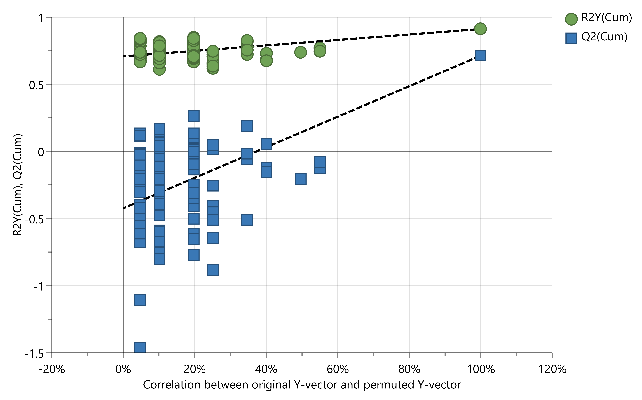

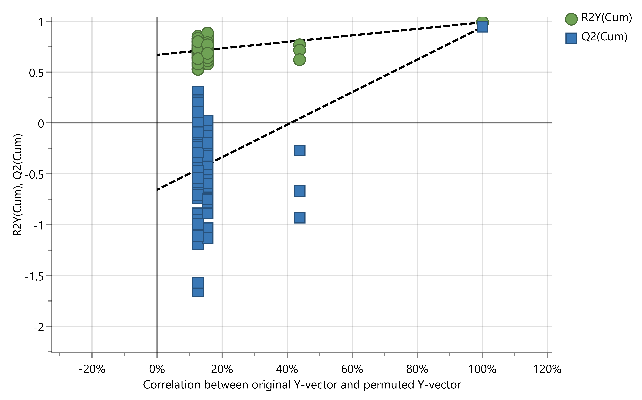


The fitness values of R2X, R2Y, and Q2 of the OPLS-DA models of each group are as follows; CON group (A1): R2X=0.526, R2Y=0.991, Q2=0.648, ILR group; (B1): R2X=0.25, R2Y=0.911, Q2=0.713; IHR group (C1): R2X=0.416, R2Y=0.988, Q2=0.942. The permutation test plots of the CON (A2), ILR (B2), and IHR (C2) group that shows the reliability of the established models:

OPLS-DA, Orthogonal partial least squares-discriminant analysis; CON, control; ILR, informed-low risk; IHR, informed-high risk

**
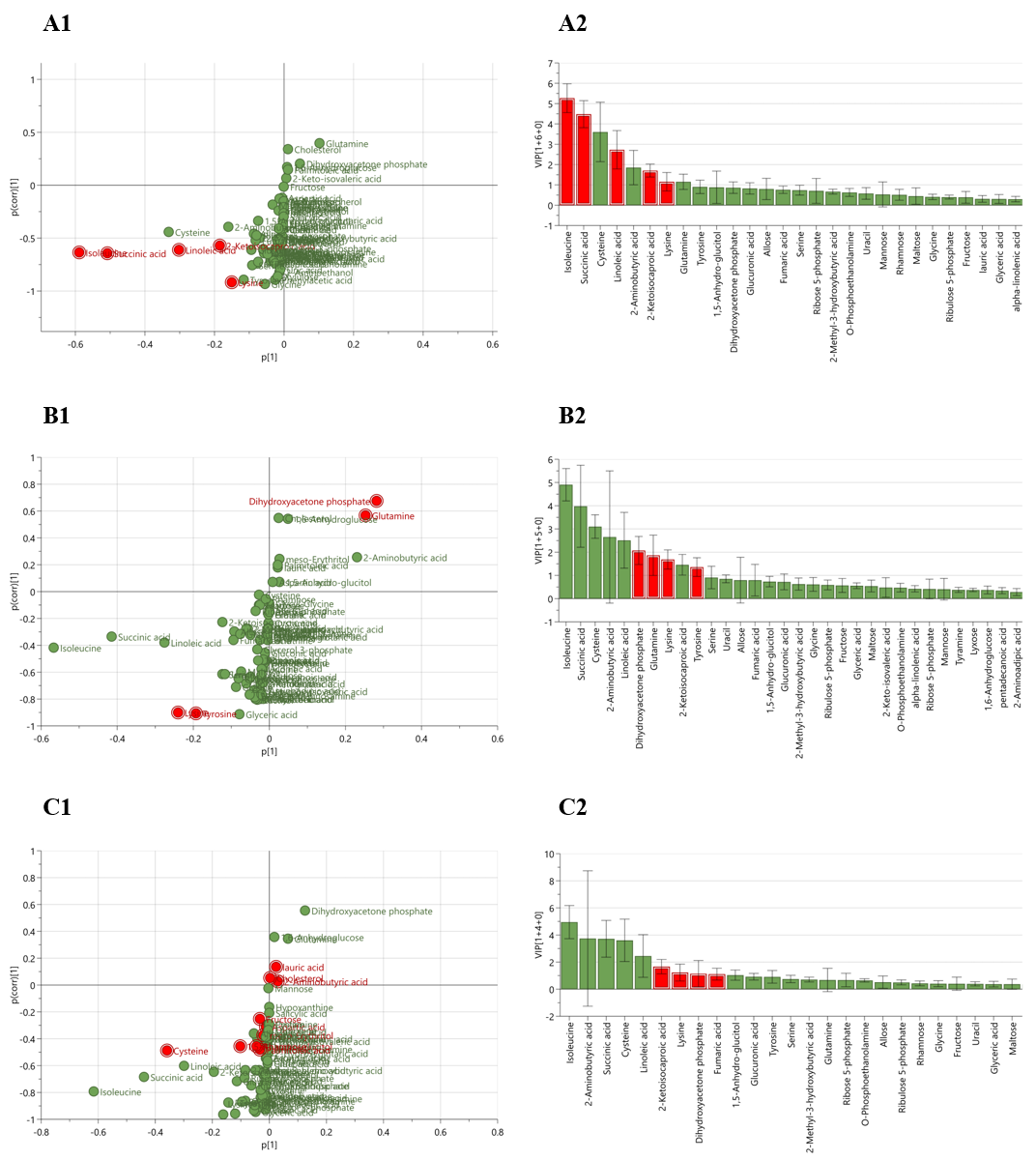
Supplementary Figure 5.** **Representative OPLS-DA S-Plots (A1, B1, and C1) and VIP plots (A2, B2, and C2) showing relative contribution of metabolomic clustering of each group**

Each point in the S-plots represents a metabolite, and the p[1] axis represents the magnitude of the spectral metabolites. Metabolites marked with red color in the S-plot and VIP plot represents strong discriminating variables, as they meet the criteria of ｜P｜≥ 0.05 and ｜P (corr)｜≥ 0.5 as well as the criteria of VIP scores > 1 and standard errors < 1.

(A) CON, control; (B) ILR, informed-low risk; (C) IHR, informed-high risk
